# Nickel nanoparticles supported on a covalent triazine framework as electrocatalyst for oxygen evolution reaction and oxygen reduction reactions

**DOI:** 10.3762/bjnano.11.62

**Published:** 2020-05-11

**Authors:** Secil Öztürk, Yu-Xuan Xiao, Dennis Dietrich, Beatriz Giesen, Juri Barthel, Jie Ying, Xiao-Yu Yang, Christoph Janiak

**Affiliations:** 1 Institut für Anorganische Chemie und Strukturchemie, Heinrich-Heine-Universität Düsseldorf, 40204 Düsseldorf, Germany; 2 State Key Laboratory of Advanced Technology for Materials Synthesis and Processing and School of Materials Science and Engineering, Wuhan University of Technology, 430070 Wuhan, China; 3 Ernst Ruska-Centrum für Mikroskopie und Spektroskopie mit Elektronen, Forschungszentrum Jülich GmbH, 52425 Jülich, Germany

**Keywords:** covalent triazine framework (CTF), electrocatalysis, nickel nanoparticles, oxygen evolution reaction, oxygen reduction reaction

## Abstract

Covalent triazine frameworks (CTFs) are little investigated, albeit they are promising candidates for electrocatalysis, especially for the oxygen evolution reaction (OER). In this work, nickel nanoparticles (from Ni(COD)_2_) were supported on CTF-1 materials, which were synthesized from 1,4-dicyanobenzene at 400 °C and 600 °C by the ionothermal method. CTF-1-600 and Ni/CTF-1-600 show high catalytic activity towards OER and a clear activity for the electrochemical oxygen reduction reaction (ORR). Ni/CTF-1-600 requires 374 mV overpotential in OER to reach 10 mA/cm^2^, which outperforms the benchmark RuO_2_ catalyst, which requires 403 mV under the same conditions. Ni/CTF-1-600 displays an OER catalytic activity comparable with many nickel-based electrocatalysts and is a potential candidate for OER. The same Ni/CTF-1-600 material shows a half-wave potential of 0.775 V for ORR, which is slightly lower than that of commercial Pt/C (0.890 V). Additionally, after accelerated durability tests of 2000 cycles, the material showed only a slight decrease in activity towards both OER and ORR, demonstrating its superior stability.

## Introduction

A worldwide increasing energy demand combined with the depletion of fossil fuels and environmental issues requires the development of new sustainable clean energy sources [1]. In many renewable energy conversion and storage systems, the oxygen evolution reaction (OER) and the oxygen reduction reaction (ORR) are two crucial processes, which require improvements through the design of efficient catalysts. Both OER and ORR suffer from slow kinetics of the four-electron transfer process [2–3]. Thus, highly efficient electrocatalysts with enhanced performance need to be developed. Noble metals (Ir, Ru) and their oxides are the current commercial electrocatalysts for the OER, whereas Pt metal is the benchmark catalyst for the ORR [4–5]. Yet, all these catalysts have drawbacks such as scarcity and high cost, which are disadvantageous for their large-scale production and application. Consequently, researchers are working on discovering and developing catalysts for OER and ORR that are metal-free or based on non-noble metals, stable and earth-abundant [6–10].

Among the transition-metal-based OER and ORR catalysts, Ni-containing catalysts are promising candidates [7,11–13]. The performance of the nickel catalysts could be further enhanced via modifications, such as the usage of carbon supports including N-doped graphene [14], active carbon [15], graphene oxide [16–17], carbon nanotubes [12,18] and covalent triazine frameworks (CTFs) [19–20].

CTFs are nitrogen-containing aromatic polymer frameworks with triazine rings, which exhibit high surface area, porosity, and thermal and chemical stability [21–22]. CTFs are promising materials for applications such as catalysts or catalyst support [23–25] and for energy storage and conversion [26–28]. CTFs can be synthesized through different methods and under different reaction conditions, which enables the control over porosity and surface area [29–32]. The nitrogen moieties within the CTFs can provide coordination anchors or support for metal species [33–34]. They allow for the stabilization of metal nanoparticles and for a good dispersion of active species that are formed upon reduction of coordinated or impregnated metal precursors while minimizing their agglomeration and leaching [35]. In the literature we can find some studies that are focused on CTFs as catalysts for ORR. In the group of Prof. Fan, Co_3_O_4_/CTF1-700-1:1 has been studied as ORR catalyst and showed a half-wave potential of 0.84 V vs a reversible hydrogen electrode (RHE) [20]. Kamiya et. al. synthesized a Pt-atom-modified CTF hybridized with conductive carbon nanoparticles and used it as an ORR catalyst [36]. The same group also produced a Cu-modified CTF hybridized with carbon nanoparticles and it showed the highest reported value among Cu-based electrocatalysts with 810 mV onset potential vs RHE for the ORR at neutral pH value [37]. In contrast, up to now there are only few studies that investigated CTFs as OER catalysts or catalyst support and the activities were far lower than that of benchmark OER catalysts [38–39]. At present, there are no reports about nickel/CTF catalysts for electrochemical OER studies, to the best of our knowledge. Although various carbon materials or nitrogen-doped carbon materials have been utilized to support nickel as electrocatalyst for the OER, novel materials with high catalytic activity and strong durability still need to be investigated (Table S3, Supporting Information File 1). In our study, by using CTFs we have the advantages of abundant aromatic nitrogen atoms with lone electron pairs, which enable a coordination of nickel, a high chemical and thermal stability arising from the covalently bonded framework as well as high surface area and large pore volume, which allow for a facile molecular transport of reactants and products.

We report a route to Ni nanoparticles supported on CTF-1 in the ionic liquid (IL) [BMIm][NTf_2_] using a microwave-assisted synthesis. The obtained material Ni/CTF-1 was investigated as a catalyst for electrochemical OER and ORR for the first time and showed a superior OER performance compared to commercial RuO_2_ under alkaline conditions and moderate ORR performance compared with commercial Pt/C.

## Results and Discussion

### Synthesis and characterization of CTF

A number of studies have already shown that CTFs as catalyst support show a better catalytic performance than other carbon-based materials [40–41]. We synthesized CTF-1 according to the literature by the ionothermal method [32,42]. Since the synthesis parameters, such as reaction temperature, affect texture, porosity and nitrogen content of the framework, two different reaction temperatures (400 and 600 °C) have been used for the synthesis (Scheme S1, Supporting Information File 1).

As expected, both CTF-1-400 and CTF-1-600 (as-synthesized) showed limited long-range order according to powder X-ray diffraction (PXRD) measurements (Figure S1, Supporting Information File 1) [32,42]. Nitrogen sorption measurements for CTF-1-400 showed a type-I isotherm with 954 m^2^/g Brunauer–Emmett–Teller (BET) surface area, whereas CTF-1-600 showed a mixture of type-I and type-IV isotherms (H2-type hysteresis) with a BET surface area of 1796 m^2^/g (Figure S2, Supporting Information File 1). The total pore volume (at *p*/*p*_0_ = 0.95) increased from 0.45 cm^3^/g for CTF-1-400 to 1.06 cm^3^/g for CTF-1-600 (see Table S2, Supporting Information File 1, for details). Elemental analyses, thermogravimetric analyses (TGA) and scanning electron microscopy (SEM) characterization data of the materials can be found in Table S1 and Figures S3–S5 in Supporting Information File 1.

### Synthesis and characterization of Ni/CTF

**Scheme 1:**
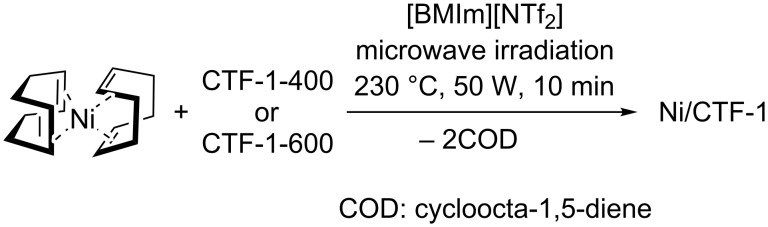
Schematic representation of Ni/CTF-1 composite synthesis via microwave-assisted thermal decomposition of Ni(COD)_2_ in the presence of CTF-1 and the ionic liquid [BMIm][NTf_2_].

For the synthesis of Ni nanoparticles (NPs) on the CTFs, the precursors bis(cycloocta-1,5-diene)nickel(0) (Ni(COD)_2_) and CTF were suspended in [BMIm][NTf_2_] by stirring under inert conditions for 12 h. The homogenized suspension was irradiated with microwaves and yielded Ni NPs immobilized on the CTFs via the decomposition of the metal precursor in the IL (Scheme 1). The composites were designated Ni/CTF-1-400-X and Ni/CTF-1-600-X, where X represents the weight percentage of nickel in the composite material based on flame atomic absorption spectroscopy (AAS). Nickel loadings of 20 to 35 wt % on CTF-1 were obtained. The initial Ni/CTF mass ratios were 1:2 and 1:1. Thus, a large fraction of the nickel precursor was indeed deposited on the CTF. The starting mass ratio of 1:2 (or 33 wt % Ni) yielded 20–22 wt % Ni/CTF-1; the ratio of 1:1 (corresponding to 50 wt % Ni) gave 33–35 wt % Ni/CTF-1. This means that only a small part of the Ni NPs remains in the IL dispersion and supports the suggested role of nitrogen atoms in the CTFs as anchor points for the Ni NPs. The obtained nickel loadings on CTF-1 are similar to what has been reported for Ni nanoparticles encased in graphitic layers (25.2 wt %), Ni encapsulated within single-layer graphene (32.8 wt %), but higher than that of nickel nanoparticles encapsulated in N-doped carbon nanotubes (14.5 wt %), and much lower than those of with N-doped carbon shells coated face-centered cubic (fcc) or hexagonal closed packed (hcp) nickel (69 and 71 wt %, respectively, see Table S3, Supporting Information File 1).

In the literature, there are various reports on Ni/carbon and Ni/N-doped carbon composites (Table S3, Supporting Information File 1). These composites are largely obtained by pyrolysis of Ni precursors or Ni-containing metal organic frameworks (MOF) with or without a nitrogen source [13,43–44]. An important step in these syntheses is high-temperature pyrolysis under inert atmosphere for a few hours. However, these methods often cannot control the nitrogen microstructure and composition. In contrast, Ni/CTF-1 is obtained in a fast and efficient microwave synthesis within 10 min from Ni(COD)_2_ and the CTF substrate in an ionic liquid. The choice of the CTF substrate enables the control over nitrogen doping by selecting appropriate aromatic nitriles as monomers [32,37,40]. Also, it has been proven that the use of CTFs as support for nanoparticles can yield an advantage in terms of metal–support interactions compared to activated carbon [40].

**Figure 1:**
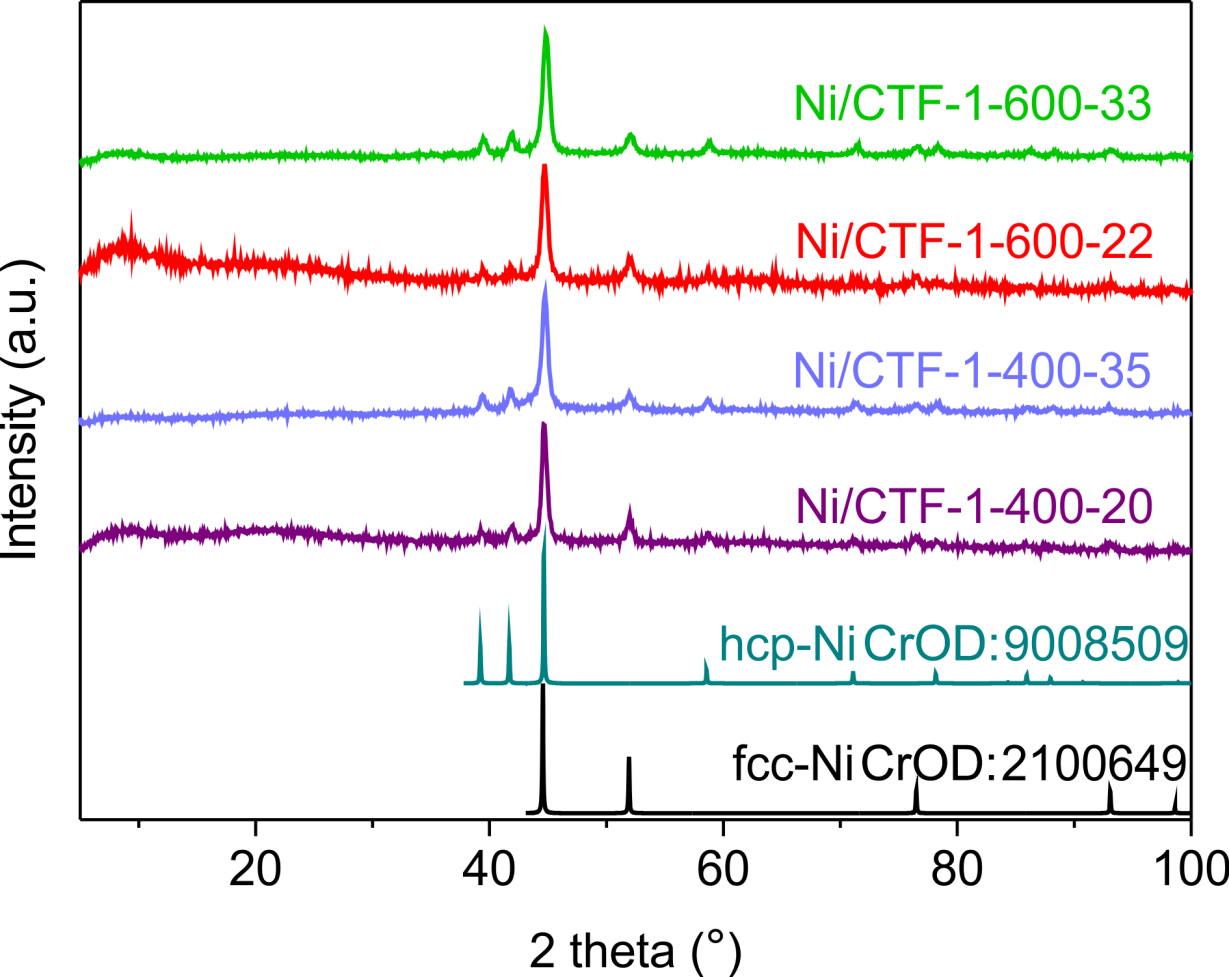
PXRD patterns of Ni/CTF-1 composite materials. The simulated diffractograms for hexagonal close-packed (hcp) and face-centered cubic (fcc) nickel are included, based on the crystallographic open database (CrOD) files.

In PXRD measurements, both cubic (fcc) and hexagonal (hcp) crystalline phases of nickel [45] were observed in all composites (Figure 1). When the nickel loading was low (20–22 wt %), the characteristic broad reflections for amorphous CTF could also be seen.

**Figure 2:**
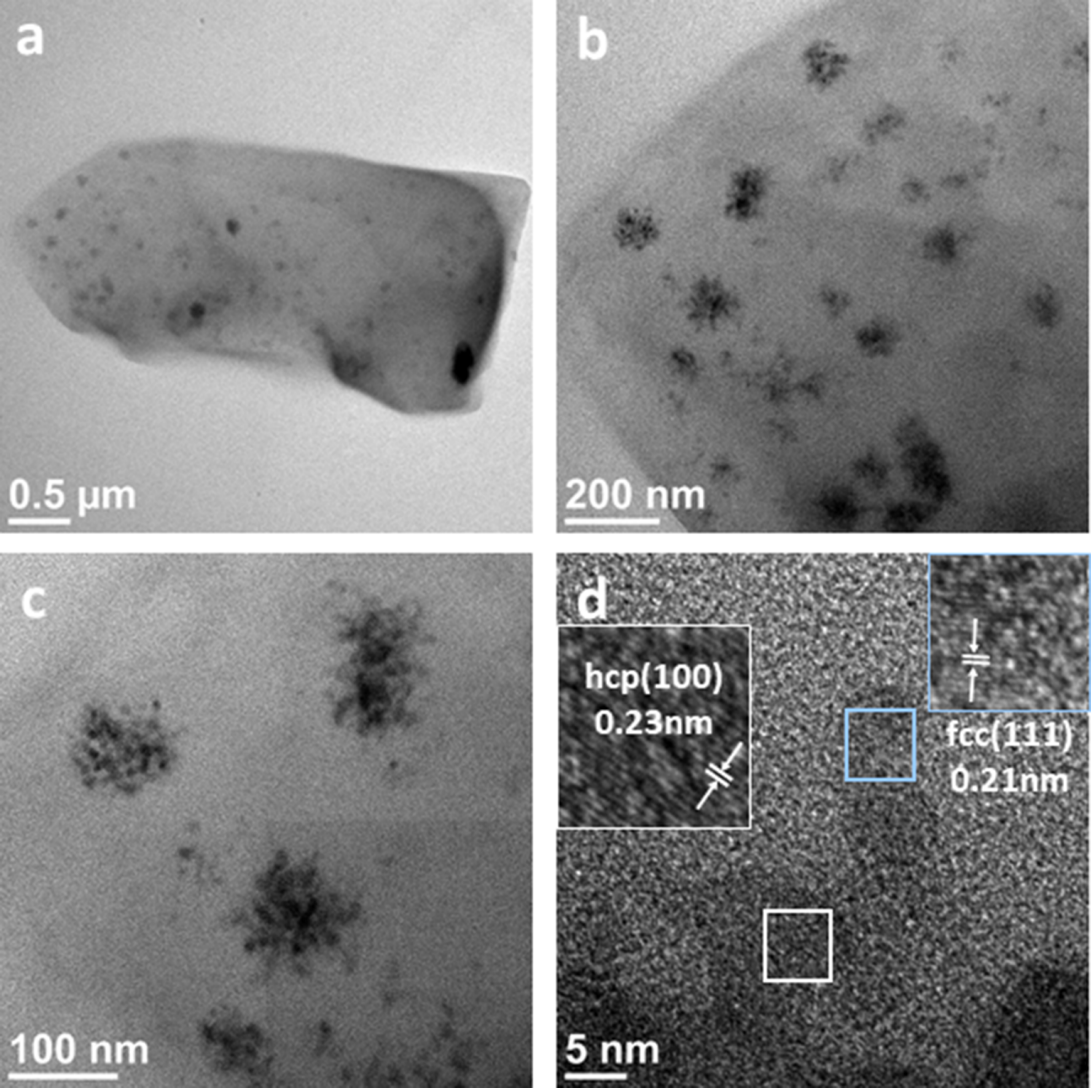
TEM images of Ni/CTF-1-600-22 showing (a) Ni nanoparticles supported on CTF, (b,c) the aggregation of the primary Ni nanoparticles and (d) the lattice planes of the Ni nanoparticles.

Size and morphology of the synthesized Ni NPs on CTFs were characterized by transmission electron microscopy (TEM) and SEM. Figure 2 shows TEM images of Ni/CTF-1-600-22 recorded at different magnifications. Ni nanoparticles supported on CTF-1 can be observed. For images of the other Ni/CTF-1 composites see Figures S6–S8 and Figure S10, Supporting Information File 1. Figure 2c shows that nickel nanoparticles with an average diameter of 10 ± 2 nm are localized on the shard-like structures of CTF and appear to form aggregates with an average diameter of 72 ± 16 nm (see Figure S9, Supporting Information File 1 for size distributions). In high-resolution TEM images of the primary small Ni NPs (Figure 2d), interplanar spacings of the lattice fringes of 0.21 nm and 0.23 nm could be measured, which corresponds to the {111} lattice spacing of face-centered cubic (fcc) Ni and the {100} lattice spacing of hexagonal close-packed (hcp) Ni, respectively. These results are in good agreement with the PXRD data of Ni/CTF-1-600-22 shown in Figure 1, where both fcc and hcp Ni were observed.

**Figure 3:**
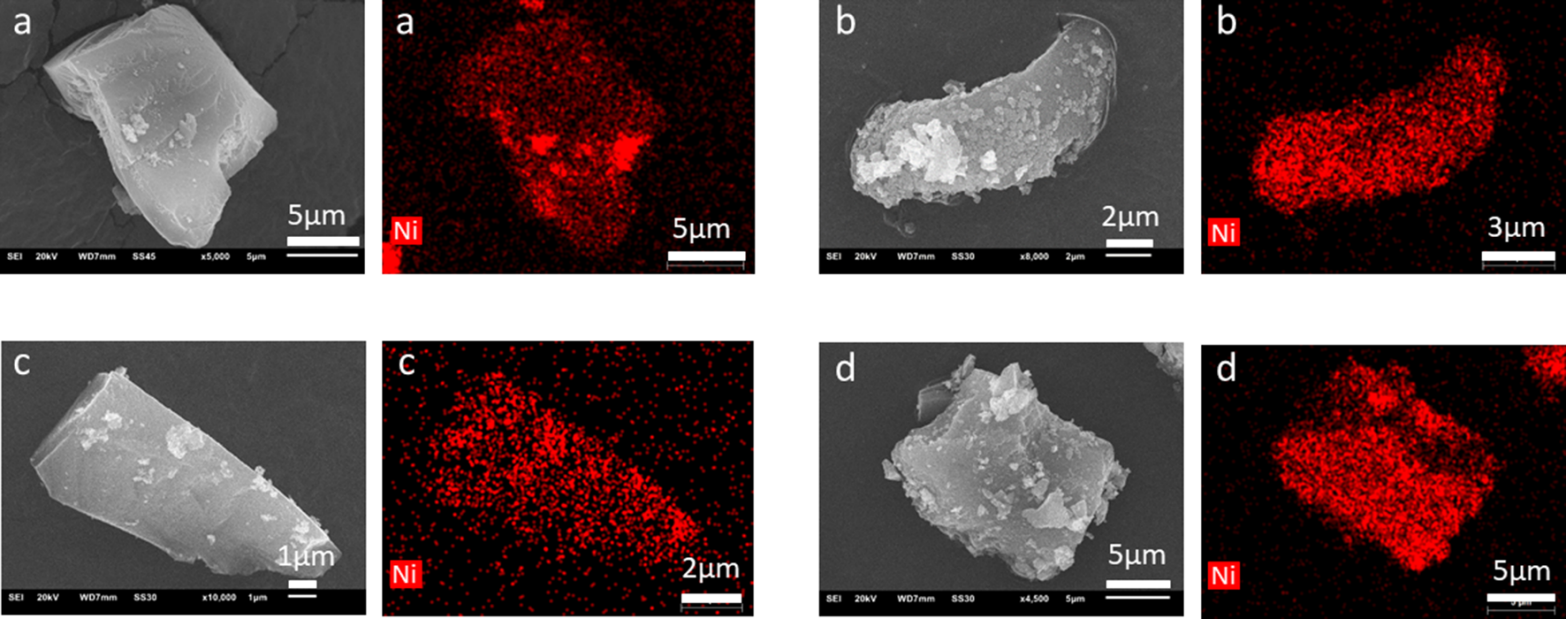
SEM images and EDX elemental mappings of Ni for (a) Ni/CTF-1-400-20, (b) Ni/CTF1-400-35, (c) Ni/CTF-1-600-22 and (d) Ni/CTF-1-600-33. Ni atoms are depicted in red.

The nickel loading on the CTF structures was further investigated by SEM-EDX elemental mapping (Figure 3), which also shows well-dispersed nickel nanoparticles on CTF. Given that nickel was detected by EDX on the whole surface of the CTF and not only on the agglomerate Ni NPs areas, further studies were performed using scanning transmission electron microscopy (STEM)-EDX elemental mapping. The element compositions of defined areas (orange square) showing both a single Ni particle and its CTF support in the background are displayed in Figure S11, Supporting Information File 1.

A possible reason why Ni NPs outside the NP agglomerates are not visible in the TEM images (Figure 2) can be either their small size or the fact that they lie between the CTF sheets (Figure S10c, Supporting Information File 1). Additional EDX studies (Figure S12, Supporting Information File 1) showed a higher content of Ni in the “brighter” agglomerates (point 1), as well as a lower but still measurable Ni count on the seemingly bare CTF shards (point 2). Thus, we conclude that nickel atoms are both found accumulated as larger NP agglomerates on the surface and as smaller nickel clusters. The smaller clusters can either reside on the surface or be included in the CTF framework. Furthermore, examination of the edges of the framework and of the nickel NPs revealed a partial oxidation. Figure S11d,f shows a correlation between Ni and O for the Ni/CTF-1-600-22 composite. This cannot be avoided, since the material was not handled under inert atmosphere (Figure S11 and Figure S13, Supporting Information File 1). The EDX point spectrum in Figure S12 for point 1 shows that the intensity of the Ni signal is stronger than the intensity of the O signal. An estimation of the atomic Ni/O ratio indicates that a significant amount of Ni at point 1 is not oxidized.

**Figure 4:**
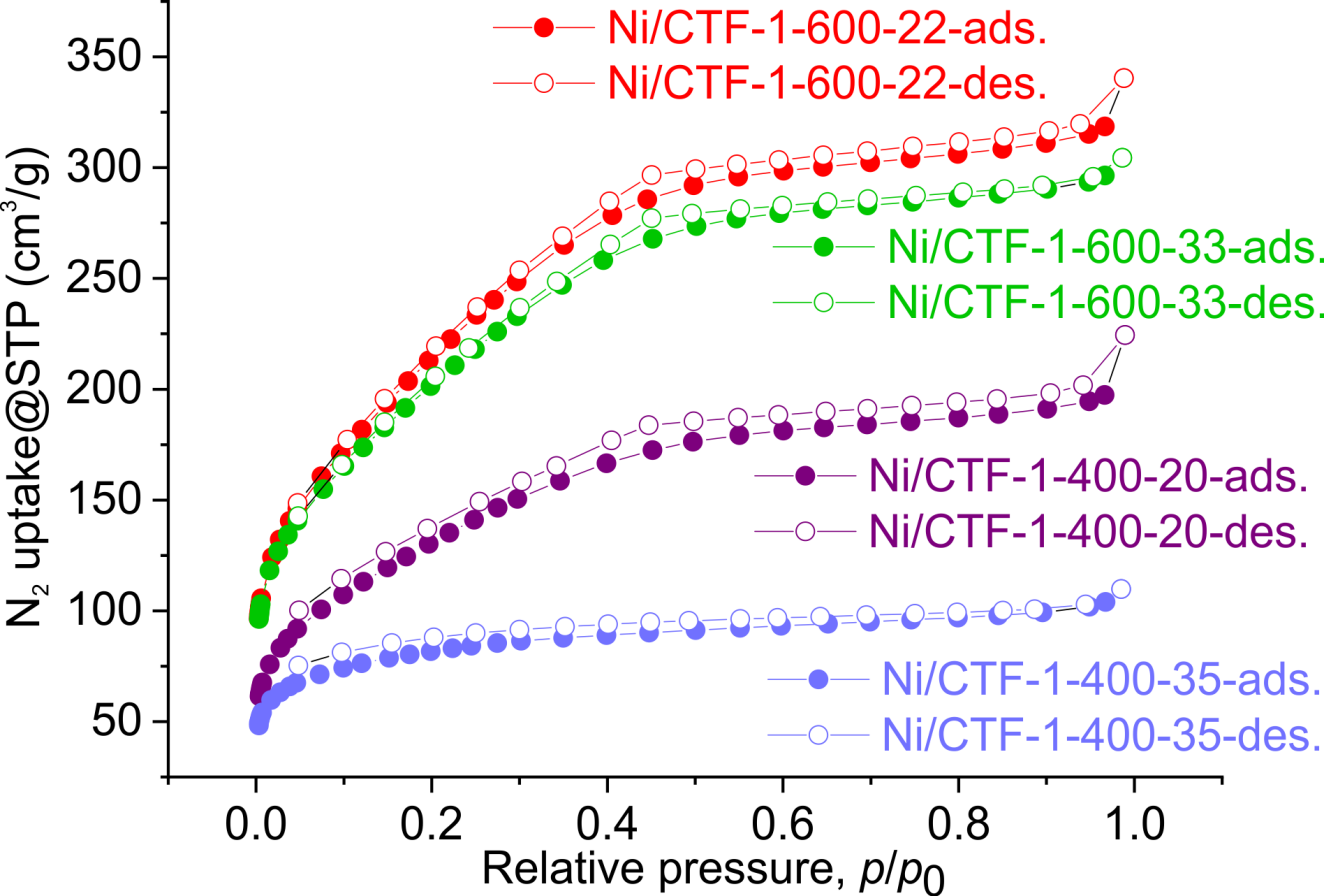
Nitrogen adsorption and desorption isotherms (at 77 K) of Ni/CTF-1 composites.

N_2_ sorption isotherms were collected to obtain information about the porosity and the BET surface area of the materials. As shown in Figure 4, the BET surface area decreases as the nickel loading increases for each CTF-1-400 and CTF-1-600 support. Ni/CTF-1-400-20 exhibits a BET surface area of 486 m^2^/g whereas Ni/CTF-1-400-35 shows a BET surface area of 300 m^2^/g. For Ni/CTF-1-600-22 and Ni/CTF-1-600-33, the BET surface area is 816 m^2^/g and 752 m^2^/g, respectively. All CTFs with Ni show a lower BET surface area and pore volume than the corresponding pristine CTF materials, which can be attributed to the incorporation of nickel into the voids of CTF-1 (Table S2, Supporting Information File 1). Still, surface area and porosity of the Ni/CTF-1 composites are high, which are important features. It is accepted that conductivity plays a more important role, yet high surface area and porosity are known to enhance the exposure of active sites and to improve the ion and charge transfer through nanochannels together with the electron-conductive medium [46]. Here, the increase of conductivity and surface area from CTF-1-400 to CTF-1-600 go in the same direction and cannot be differentiated regarding their role in improving the activity of the CTF-1-600.

**Figure 5:**
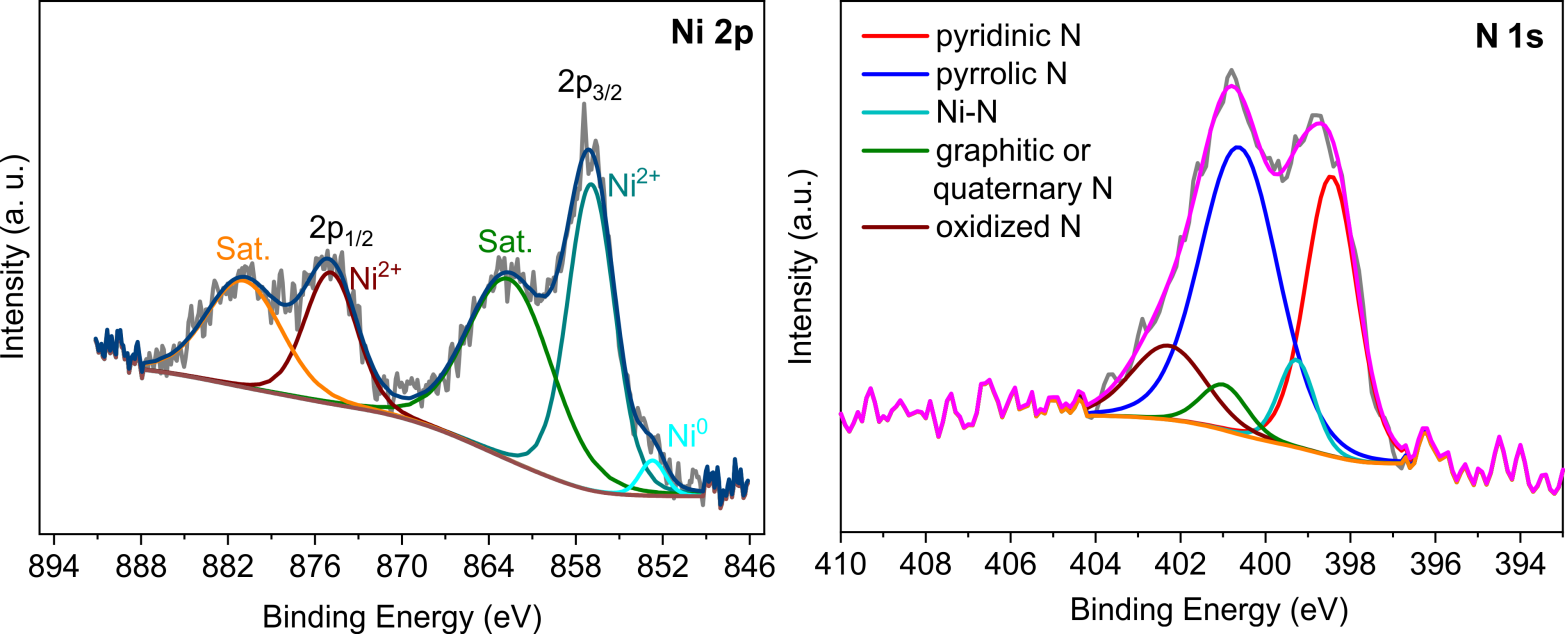
XPS measurements of Ni/CTF-1-600-22 with deconvoluted Ni 2p (left) (Sat. = satellite) and N 1s spectra (right).

X-ray photoelectron spectroscopy (XPS) provides information about the chemical composition and chemical state of elements. The Ni 2p and N 1s spectra of the materials are shown in Figure 5 and Figure S15–S18, Supporting Information File 1. The Ni 2p spectrum of Ni/CTF-1-600-22 consists of the main peak of Ni^0^, of which only the more intense 2p_3/2_ peak at 852.7 eV is visible, because of the overall small amount of Ni^0^. The Ni spectrum is dominated by the two main peaks of Ni^2+^ at 856.9 and 874.9 eV. The two peaks at 862.5 and 880.8 eV are satellite peaks of Ni^2+^. Ni always shows strong satellites about 6 eV above the main electronic lines [47]. In composite materials, Ni^2+^ can arise from the combination of nickel coordinated with nitrogen and from the oxidation/hydroxylation of nickel (since the samples need to be briefly handled in air to be introduced into the XPS instrument).

Deconvolution of the N 1s XPS spectrum of Ni/CTF-1-600-22 reveals five peaks at about 398.5, 399.3, 400.6, 401.2 and 402.3 eV, which can be assigned pyridinic nitrogen, Ni-coordinated nitrogen, pyrrolic nitrogen, graphitic or quaternary nitrogen and oxidized nitrogen, respectively [26,48]. The formation of pyridinic N and graphitic or quaternary N have been demonstrated to improve the activity of N-modified carbon-based materials such as N-doped ordered porous carbon and N-doped carbon nanotubes [49–50]. According to our evaluation of the XPS data, 8 atom % N is involved in bonding to Ni for Ni/CTF-1-400-20, whereas 7 atom % N is involved in bonding to Ni for Ni/CTF-1-600-22.

### Electrochemical catalysis

**Figure 6:**
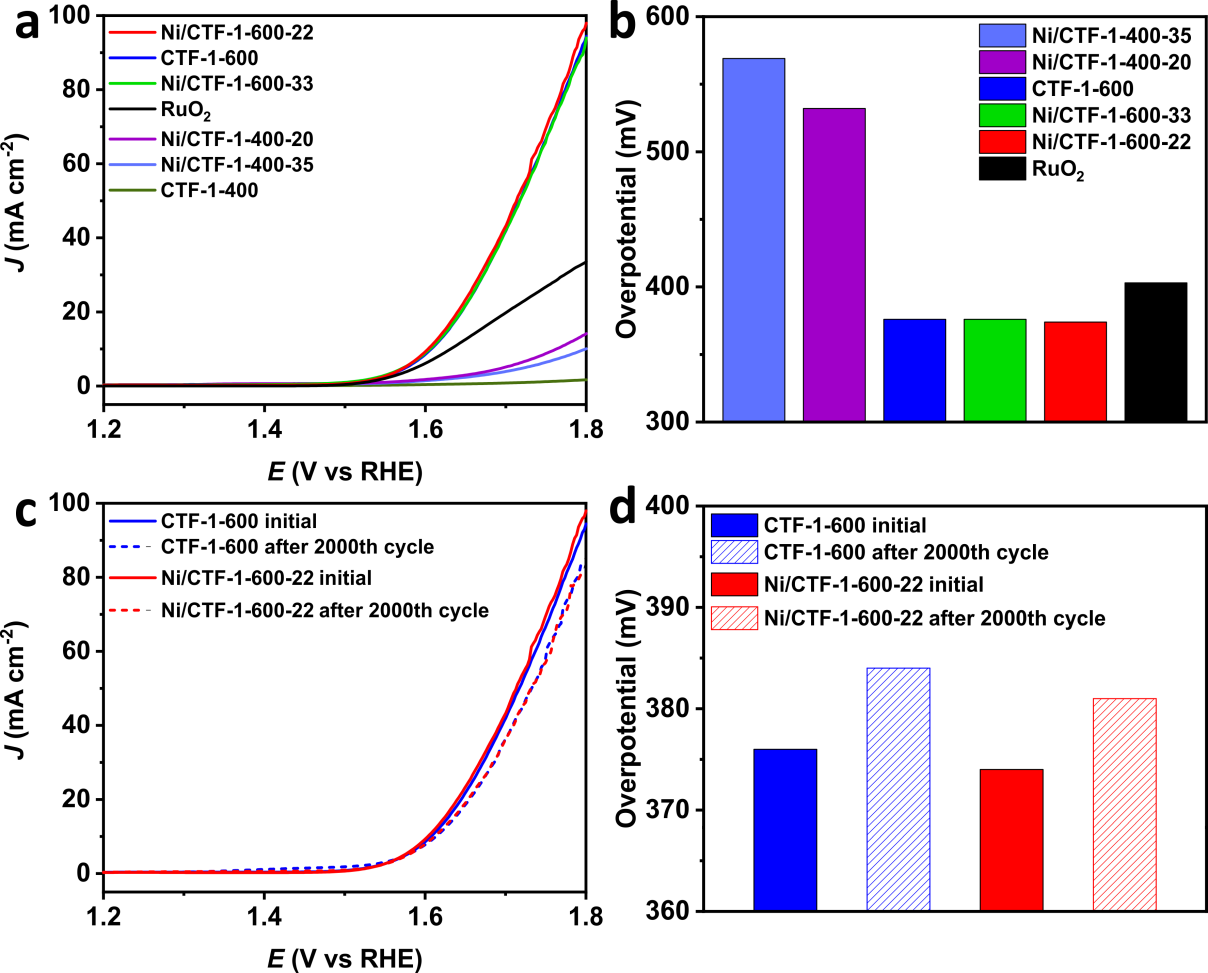
(a) OER polarization curves of various materials, (b) overpotential values calculated from (a); (c) OER polarization curves for CTF-1-600 and Ni/CTF-1-600-22 after 2000 cycles, (d) overpotential values calculated from (c).

In order to investigate the activity of the synthesized materials in the OER, rotating disc electrode (RDE) experiments were conducted in 1 mol/L KOH solution in a three-electrode cell. Figure 6a shows the OER polarization curves of the catalysts measured with a sweep rate of 5 mV/s at room temperature. We note that there should be a nickel oxidation process visible before the onset of the OER. Yet, the oxidation process of Ni for OER may be not evident [51–52]. Further, from XPS (Figure 5 and Supporting Information S15–S18, Supporting Information File 1) we can confirm the presence of Ni species on the surface of the composite materials but it is evident that nickel nanoparticles are already oxidized due to the handling of the sample in air.

**Figure 7:**
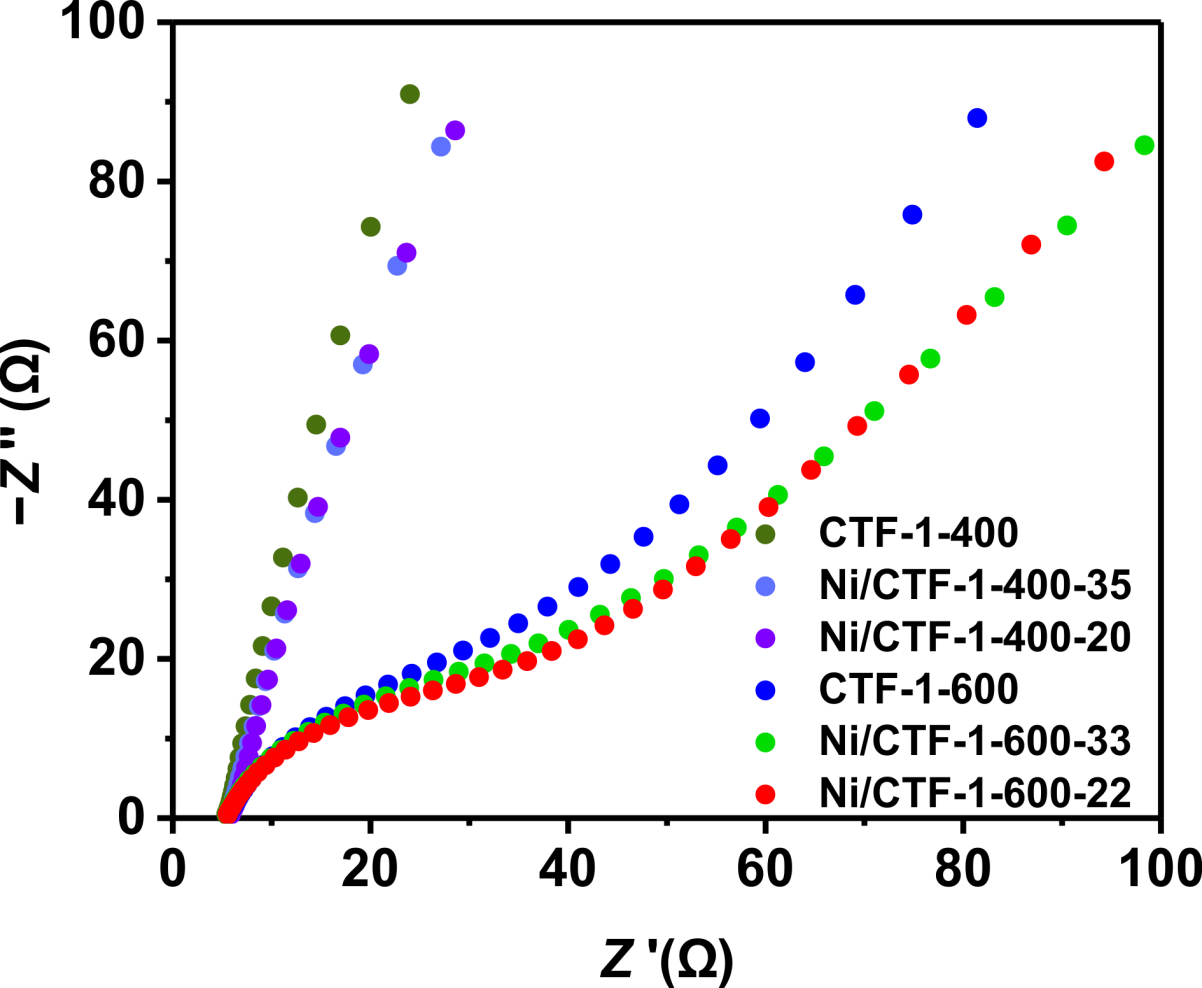
Nyquist plots recorded in 1 mol/L KOH solution.

The materials based on CTF-1-600 show a significantly higher OER activity than those based on CTF-1-400. Pristine CTF-1-400 showed almost no activity towards OER, whereas Ni/CTF-1-400-35 and Ni/CTF-1-400-20 showed higher activity with a required overpotential of 569 and 532 mV to reach 10 mA/cm^2^, respectively. Nevertheless, all three samples showed much lower catalytic OER activity than the benchmark RuO_2_ catalyst, which requires 403 mV under the same conditions. In contrast, pure CTF-1-600, Ni/CTF-1-600-33 and Ni/CTF-1-600-22 all show essentially identical OER activities with overpotentials of 376 mV for CTF-1-600, 374 mV for Ni/CTF-1-600-22 and 376 mV for Ni/CTF-1-600-33, which is better than the 403 mV for RuO_2_ (Figure 6b). Moreover, the Tafel plots confirm the identical OER behavior of pure CTF-1-600, Ni/CTF-1-600-33 and Ni/CTF-1-600-22 (Figure S19, Supporting Information File 1). The very similar behavior among the CTF-1-600 series also suggests that the presence of Ni has no significant effect on CTF-1-600 for OER. Bare CTF-1-600 is found to be a good OER catalyst to begin with. At the same time, bare nickel was already shown not to be a good OER catalyst, having an overpotential of around 390 mV at 10 mA/cm^2^ (vs RHE) or showing a current density of 0.25 mA/cm^2^ at 1.70 V (vs RHE) [53–54]. The OER activity of Ni oxide and hydroxides in combination with carbon materials is ambiguous. NiO nanoparticles have an overpotential of 331 mV at 10 mA/cm^2^ (vs RHE) [55], which increases to 422 mV at 10 mA/cm^2^ (vs RHE) for a NiO nanoarray grown on carbon cloth [56]. Similarly, Ni(OH)_2_ nanoparticles have an overpotential of only 299 mV to reach 10 mA/cm^2^ (vs RHE) [55]. But this value increases to 462 mV for Ni(OH)_2_ grown on carbon cloth [56]. Thus, the high electrocatalytic activity of the bare CTF-1-600 support is adversely affected by the admixture of Ni species with low activity in the composite materials. The better OER performance of CTF-1-600 over the CTF-1-400 materials is attributed to the better conductivity of the former (as given by the Nyquist plot in Figure 7) and its faster ion and charge transfer together with its higher porosity (Table S2, Supporting Information File 1). It is therefore understandable that the less conductive CTF-1-400 shows improved OER characteristics after the deposition of Ni.

Although Ni/CTF-1-600-22 is not the best catalyst compared to other carbon-supported nickel electrocatalysts (Table S3, Supporting Information File 1), its performance is better than many of these catalysts and its synthesis does not require special techniques or sophisticated equipment. The modifiable nitrogen functionalities enable stabilization and dispersion of metal sites throughout the support. The high chemical and thermal stability of the CTF support is another advantage, as this is often a problem of many other catalysts. CTF-1-600, as a metal-free electrocatalyst, showed better performance than N-doped carbon nanomaterials, which required an overpotential of 0.38 V vs RHE at 10 mA/cm^2^ [57], better performance than N-doped carbon sheets requiring 0.41 V vs RHE [58] and better performance than nitrogen-doped graphene/carbon nanotube hybrids requiring an overpotential of 0.4 V vs RHE at 10 mA/cm^2^ [59]. In comparison with the literature, CTF-1-600 and Ni/CTF-1-600-22 appear to be highly active OER electrocatalysts (cf. Table S3, Supporting Information File 1).

Accelerated durability tests (ADTs) for cyclic potential sweeps were carried out for CTF-1-600 and Ni/CTF-1-600-22 in order to examine the durability of the catalysts. As shown in Figure 6c, the slope of CTF-1-600 and Ni/CTF-1-600-22 only shows a slight change after 2000 cycles. After 2000 cycles, the overpotential of CTF-1-600 changed from 376 to 384 mV, while the overpotential of Ni/CTF-1-600-22 changed from 374 to 381 mV (Figure 6d). The small change in the overpotential reveals the superior stability of these two materials.

**Figure 8:**
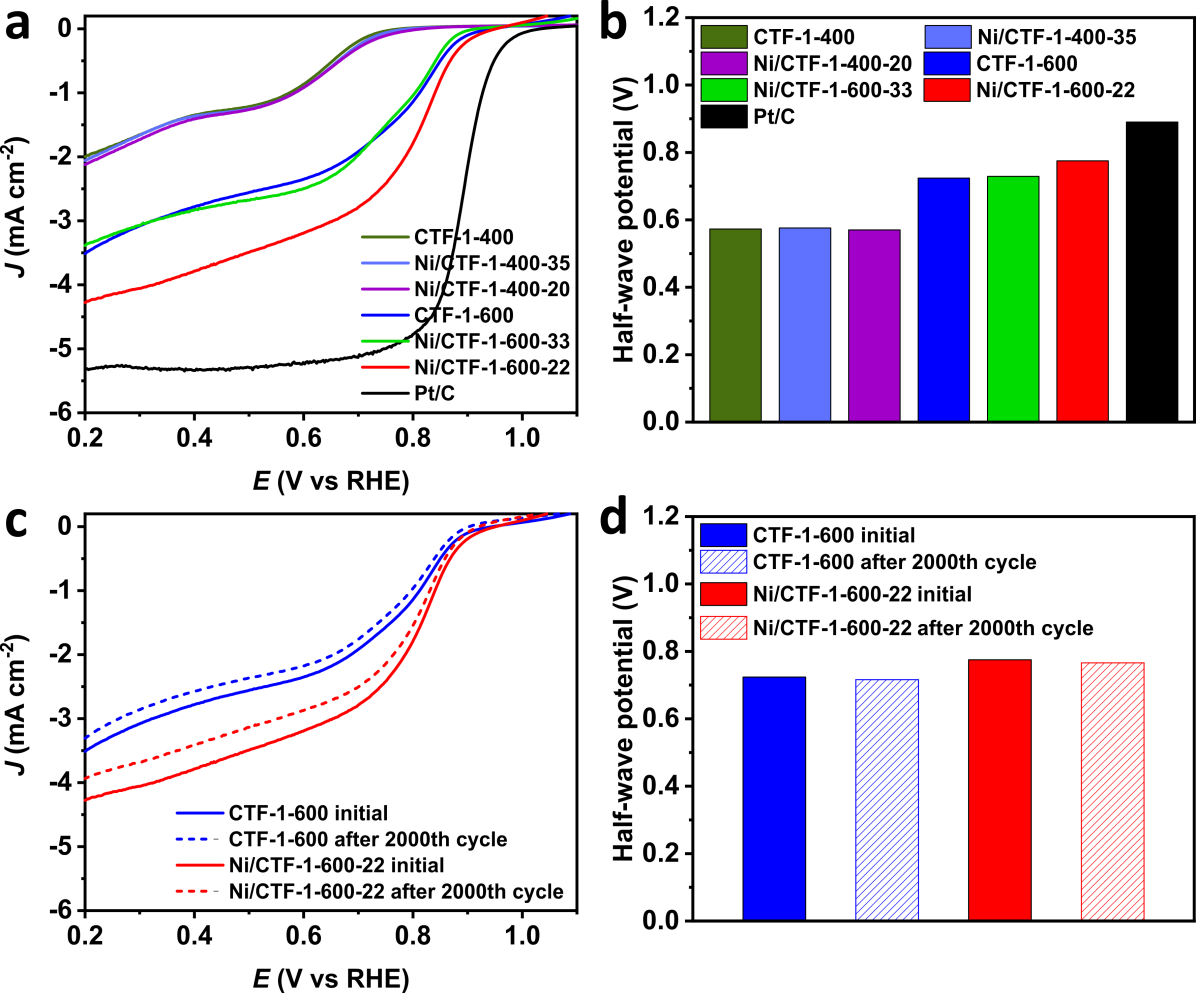
(a) ORR polarization curves of various materials, (b) half-wave potential values calculated from (a); (c) ORR polarization curves for CTF-1-600 and Ni/CTF-1-600-22 after 2000 cycles, (d) half-wave potential values calculated from (c).

To examine the electrocatalytic ORR activity of the materials, polarization curves were collected in O_2_-saturated 1 mol/L KOH solution with a sweep rate of 10 mV/s at room temperature. The half-wave potentials are shown in Figure 8a. CTF-1-400, Ni/CTF-1-400-20 and Ni/CTF-1-400-35 show similar ORR polarization curves and their half-wave potentials of 0.573 V, 0.570 V and 0.576 V, respectively, are far smaller than those of the CTF-1-600 samples. The very similar potentials of the CTF-1-400 materials suggest that the presence of Ni has no significant effect on CTF-1-400 for ORR. The better ORR performance of CTF-1-600 over the CTF-1-400 materials is attributed to the better conductivity of the former (as obtained from the Nyquist plot in Figure 7).

The half-wave potential of Ni/CTF-1-600-22 (0.775 V) was larger than that of CTF-1-600 (0.724 V) and that of Ni/CTF-1-600-33 (0.729 V), indicating a faster dynamic process regarding ORR activity for Ni/CTF-1-600-22. Compared to the half-wave potential of 0.890 V for commercial Pt/C, Ni/CTF-1-600-22 showed the best ORR performance among all tested Ni/CTF-1 and CTF-1 catalysts (Figure 8b). The Tafel plots also show that Ni/CTF-1-600-22 exhibits the best ORR activity and the performance (among Ni/CTF-1 materials) closest to commercial Pt/C (Figure S19, Supporting Information File 1). The half-wave potential of Ni/CTF-1-600-22 (0.775 V) is comparable to those given in the literature, such as nickel encapsulated in nitrogen-doped carbon nanotubes with a half-wave potential of 0.73 V (vs RHE) [60], nickel nanoparticles encased in graphitic layers with a half-wave potential of 0.78 V (vs RHE) [61] and nickel/nitrogen co-doped carbon nanocubes with a half-wave potential of 0.835 V (vs RHE) [62]. ADTs were performed to evaluate the stability of CTF-1-600 and Ni/CTF-1-600-22. The slope of both materials after 2000 cycles is shown in Figure 8c. The half-wave potential of CTF-1-600 dropped slightly from 0.724 V to 0.716 V, while the half-wave potential of Ni/CTF-1-600-22 dropped from 0.775 V to 0.766 V, indicating a good stability of both materials (Figure 8d).

The reason for the good electrochemical ORR performance of Ni/CTF-1-600-22 was investigated by electrochemical impedance spectroscopy (EIS). As shown in Figure 7, all CTF-1-600 materials exhibited a higher conductivity than CTF-1-400. This could be ascribed to the higher graphitization degree achieved through the higher synthesis temperature. Moreover, for the CTF-1-600 samples, the conductivity shows a trend of Ni/CTF-1-600-22 > Ni/CTF-1-600-33 > CTF-1-600, which is in accordance with the ORR results. In other words, the highest conductivity of Ni/CTF-1-600-22 coincided with the highest electrochemical ORR activity among all CTF species. ORR and OER are two reverse reaction sequences. In alkaline electrolyte, the mechanism of OER/ORR goes through the following elementary steps where S* is an active surface site or a surface-bound/adsorbed intermediate species, such as S–OH*, S–O* (in the literature often only * is used) [63]:


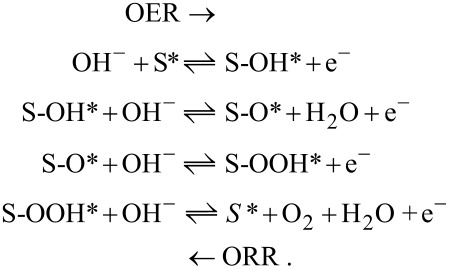


The OER proceeds through the formation of S-OH* and the ORR proceeds through the formation of S-OOH* in the reverse direction. Considering the mechanisms, the ORR and OER activity is limited by different rate-limiting steps for each reaction. The OER activity is limited by the S-O* and S-OOH* formation steps, whereas the ORR activity is limited by the S-OH* and O_2_ reduction steps. In this regard, ORR and OER catalysts need to have different binding energies for intermediates for optimum activity. In addition to this, metal species undergo oxidation at the high positive potentials required for OER, which gives a positively charged more oxidized surface that is different than under reductive ORR conditions. Consequently, the best ORR catalyst may not simultaneously be the best OER catalyst [63].

For a Co_3_O_4_/CTF700-1:1 composite it has been founded that it exhibits improved ORR activity (half-wave potential of 0.84 V) compared to pure CTF700 and Co_3_O_4_ nanoparticles. The amount of Co_3_O_4_ in the composite material played an important role since it changed the activity of the composite but no activity trend related to the different used amounts was observed [20]. We observed a better activity of Ni/CTF-1-600-22, which means that a fraction of 22 wt % Ni is apparently more suitable than the 33 wt % in Ni/CTF-1-600-33.

In the literature, Ni(OH)_2_/graphene oxide showed a significant enhancement of the ORR activity compared to unsupported Ni(OH)_2_ and graphene oxide alone. The hybrid material Ni(OH)_2_/graphene oxide has an onset potential of −0.17 V vs Ag/AgCl for ORR, which is 80 to 100 mV more positive than the corresponding onset potentials of unsupported Ni(OH)_2_ (−0.25 V vs Ag/AgCl) and exfoliated graphite oxide sheets (−0.27 V vs Ag/AgCl) [64]. In another study, Ni-N/C (nickel nanoparticles/amino-N-doped carbon) exhibited an onset potential of 0.88 V vs RHE for the ORR [65] and showed a better activity than the pure amino-N-doped carbon with an onset potential of 0.82 V vs RHE [66]. Consequently, it is expected that nickel species improve the ORR activity of the catalysts.

## Conclusion

We produced CTF-1-400 and CTF-1-600 (400 and 600 being the synthesis temperature in °C) to study them as direct electrocatalysts and as supports for nickel nanoparticles to give Ni/CTF-1 materials. The latter were also tested as electrocatalysts for OER and ORR. As a result of the different synthesis temperatures, different properties in CTF-1 were obtained. The CTF-1-600 material outperformed the less conductive CTF-1-400 material and the benchmark RuO_2_ (403 mV) by reaching 10 mA/cm^2^ with an overpotential of 374 mV. It also showed high stability. The CTF-based materials were also investigated for ORR and Ni/CTF-1-600-22 with 22 wt % Ni showed the best performance with a half-wave potential of 0.775 V, reaching the performance closest to the benchmark Pt/C catalyst, which shows a half-wave potential of 0.890 V under the same working conditions. The high electrochemical performance of Ni/CTF-1-600-22 can be traced to the best conductivity among all the CTF-based electrocatalysts as investigated by EIS tests. Consequently, we believe that CTFs are potential candidates for electrochemical OER and offer room for improvement. In the future, we anticipate that this study should inspire further investigations on CTF materials for electrocatalytic applications.

## Experimental

### Materials

Bis(cycloocta-1,5-diene)nickel(0) (Ni(COD)_2_), 1,4 dicyanobenzene (98%) and 1-chlorobutane (>99%) were obtained from Sigma Aldrich, ZnCl_2_ (>98%) from Alfa Aesar and bis(trifluoromethane)sulfonimide lithium salt (99%) from ABCR. All materials were used without further purification. 1-Methylimidazole (>99%) was obtained from Sigma Aldrich, purified via fractional distillation and dried over molecular sieves for several days. Water was purified using the Millipore^®^ system. The ionic liquid (IL) 1-butyl-3-methylimidazolium bis(trifluoromethylsulfonyl)imide ([BMIm][NTf_2_]) was synthesized in two steps following a literature procedure [67]. The anion purity of IL by ion chromatography was found to be above 99% and the water content of the IL by Karl-Fischer titration was less than 10 ppm.

### Methods

Powder X-ray diffraction (PXRD) patterns were obtained at ambient temperature on a Bruker D2 Phaser powder diffractometer with a flat rotating silicon, low-background sample holder, at 30 kV, 10 mA for Cu Kα radiation (λ = 1.5418 Å). The diffractograms were analyzed with Match 3.11 software. All samples were measured between 5° and 100° 2θ with a scan speed of 2 s/step and 0.057° (2θ) step size. Nitrogen sorption measurements were performed with a Nova 4000e from Quantachrome at 77 K and evaluated with the NovaWin 11.03 software. The materials were first degassed under vacuum (<10^−2^ mbar) at 120 °C overnight. The Brunauer–Emmett–Teller (BET) surface areas were calculated from five adsorption points in the range of *p*/*p*_0_ = 0.02–0.1 for CTF-1-400 and Ni/CTF-1-400-20, of *p*/*p*_0_ = 0.1–0.3 for Ni/CTF-1-400-35 and of *p*/*p*_0_ = 0.1–0.2 for CTF-1-600 and its corresponding composites. The pore size distribution was derived by NLDFT calculations based on N_2_ at 77 K on carbon with slit pores. Thermogravimetric analysis (TGA) was done with a Netzsch TG 209 F3 Tarsus device equipped with an Al crucible applying a heating rate of 10 K/min under inert atmosphere. Elemental analyses (CHN) were performed with a Perkin Elmer 2400 apparatus. Flame atomic absorption spectroscopy (AAS) for the determination of the metal content was conducted with a Vario 6 from Analytic Jena. For AAS the sample was treated with aqua regia. Ion chromatography (IC) measurements were performed with a Dionex ICS 1100 instrument with an IonPac AS 22column, combined with suppressed conductivity detection. Karl-Fischer titration (KFT) was carried out with an ECH/ANALYTIK JENA AQUA 40:00 Karl Fischer titrator. A Carbolite Gero tube furnace has been used for the CTF synthesis. Scanning electron microscopy (SEM) images were recorded with a Jeol JSM-65 10 LV QSEM advanced electron microscope with a LaB_6_ cathode at 5–20 keV and a Bruker Xflash 410 silicon drift detector for energy-dispersive X-ray spectrometric (EDX) elemental composition analysis. M/CTF-IL suspension samples for transmission electron microscopy (TEM) were dripped on a carbon-coated copper grid and excess IL was washed off three times with acetonitrile and left to dry. Images were recorded on a FEI Tecnai G2 F20 electron microscope operated at 200 kV accelerating voltage equipped with a Gatan UltraScan 1000P detector [68]. Scanning transmission electron microscopy (STEM) images and EDX elemental mapping were conducted with the same instrument. High-resolution TEM images were recorded with an FEI Titan 80-300 transmission electron microscope [69] operated at 300 kV accelerating voltage. The microscope is equipped with an image CS corrector and a 2k × 2k GATAN UltraScan 1000 CCD. Nanoparticle size and size distribution were determined using the Digital Micrograph software from Gatan analyzing over 100 particles.

X-ray photoelectron spectroscopy (XPS) was performed using a ULVAC-PHI VersaProbe II microfocus X-ray photoelectron spectrometer. The spectra were recorded using a polychromatic Al Kα X-ray source (1486.8 eV). The C 1s orbital with a binding energy of 284.8 eV was taken as a reference for the evaluation of the spectra. CasaXPS, version 2.3.19PR1.0, copyright 1999-2018 Casa Software Ltd. program was used for the fit of the experimental XP spectra.

### Synthesis of CTF-1

CTF-1-400 and CTF-1-600 were synthesized by ionothermal reaction at 400 and 600 °C, respectively, according to the literature [32,42]. For the synthesis of CTF-1-400, 1.28 g (10 mmol, 1 equiv) of dicyanobenzene (DCB) and 6.80 g (50 mmol, 5 equiv) anhydrous ZnCl_2_ were mixed in a Duran glass ampoule under inert conditions. The ampoule was evacuated, flame-sealed and heated in a tube oven at 400 °C for 48 h. After the ampoule was cooled down to ambient temperature, it was opened and the black solid product was ground. The product was washed first with 200 mL Millipore water for 72 h. After isolation of the product by filtration, it was washed with 200 mL diluted hydrochloric acid (HCl) (2 mol/L) for 24 h. The washing process was further continued with millipore water (3 × 75 mL), tetrahydrofuran (THF) (3 × 75 mL) and acetone (3 × 75 mL). The resulted product was dried under high vacuum at 120 °C overnight. The same procedure has been followed for CTF-1-600 by first heating to 400 °C for 40 h and subsequently to 600 °C for 20 h.

### Synthesis of Ni/CTF-1 in [BMIm][NTf_2_]

Ni(COD)_2_ (23.4 mg, 0.085 mmol or 46.8 mg, 0.17 mmol) and 10 mg of CTF-1-400 or CTF-1-600 were stirred in 1 g of IL in a microwave tube at room temperature and in a glovebox for 12 h. The mass of the nickel precursor was set to yield 0.5 or 1.0 wt % metal nanoparticles in IL, whereas 1.0 wt % CTF was used for all syntheses in IL dispersions. This dispersion was placed in a microwave (CEM Discover) and irradiated with a power of 50 W to 230 °C for 10 min. The volatiles from the Ni/CTF-1 product were removed under vacuum and then the product was handled in air, washed with acetonitrile (3 × 4 mL) centrifuged (6000 rpm), and then dried under vacuum. All reactions and the analysis of the products by PXRD have been repeated several times in order to confirm the reproducibility. The obtained materials were designated as Ni/CTF-1-400-X and Ni/CTF-1-600-X, where X represents the weight percentage of nickel in the composite materials according to AAS.

### Electrochemical measurements

A three-electrode cell was used for the electrochemical measurements on a Autolab working station from Metrohm, Switzerland. Typically, a Ag/AgCl electrode (with saturated KCl solution) was used as a reference electrode, a carbon rod was used as a counter electrode, and a glassy-carbon rotating disk electrode (RDE, diameter: 5 mm, area: 0.196 cm^2^) was used as the working electrode. The loading amount of all catalysts was 0.255 mg/cm^2^. The OER measurements were carried out in 1 mol/L KOH using the glassy-carbon RDE at a rotation rate of 1600 rpm with a 5 mV/s sweep rate. The accelerated durability tests (ADTs) for OER were performed in 1 mol/L KOH solution with cyclic potential sweeps between 1.23 and 1.53 V vs reversible hydrogen electrode (RHE) at a 100 mV/s sweep rate for 2000 cycles. The ORR measurements were carried out in 1 mol/L O_2_-saturated KOH solution under O_2_ flow using the glassy-carbon RDE at a rotation rate of 1600 rpm with a 10 mV/s sweep rate. The ADTs for ORR were performed in 1 mol/L KOH solution under air with cyclic potential sweeps between 0.6 and 1.1 V versus RHE at a 50 mV/s sweep rate for 2000 cycles. The electrochemical impedance spectroscopy (EIS) measurements were performed in 1 mol/L KOH, in a frequency range of (0.1–1) × 10^5^ Hz and a small sine-wave distortion (AC signal) of 10 mV amplitude. All potentials were converted to values with reference to RHE.

## Supporting Information

File 1 Additional experimental data.
