# Supplementary material for: Nickel nanoparticles supported on a covalent triazine framework as electrocatalyst for oxygen evolution reaction and oxygen reduction reactions
Source: Beilstein J Nanotechnol. 2020 May 11;11:770–81. doi: 10.3762/bjnano.11.62 (PMC7237812; doi:10.3762/bjnano.11.62)
Supplement: File 1 — Additional experimental data. [file Beilstein_J_Nanotechnol-11-770-s001.pdf]

BEILSTEIN JOURNAL OF NANOTECHNOLOGY

Supporting Information

for

Nickel nanoparticles supported on a covalent triazine framework as electrocatalyst for oxygen evolution reaction and oxygen reduction reactions

Secil Öztürk, Yu-Xuan Xiao, Dennis Dietrich, Beatriz Giesen, Juri Barthel, Jie Ying, Xiao-Yu Yang and Christoph Janiak

Beilstein J. Nanotechnol. **2020,** *11,* 770–781. doi:10.3762/bjnano.11.62

Additional experimental data

License and Terms: This is a supporting information file under the terms of the Creative Commons Attribution License (http://creativecommons.org/licenses/by/4.0). Please note that the reuse, redistribution and reproduction in particular requires that the authors and source are credited.
The license is subject to the *Beilstein Journal of Nanotechnology* terms and conditions: (https://www.beilstein-journals.org/bjnano)

Synthesis and characterization of CTFs

CTF-1-400 was synthesized mixing 1,4-dicyanobenzene (DCB) and zinc chloride ($ZnCl_2$) with a 1:5 monomer-to-salt ratio in a sealed glass ampoule at 400 °C for 48 h. CTF-1-600 was synthesized by the same route as CTF-1-400 except that the temperature was first held at 400 °C for 40 h and then at 600 °C for 20 h.

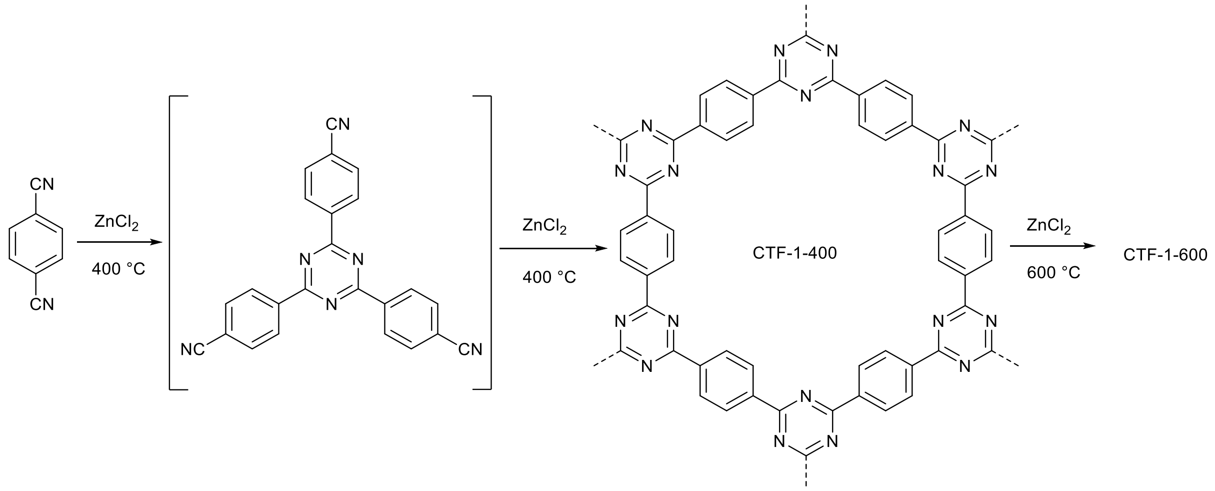

Scheme S1: Idealized schematic formation of CTF-1-400/600 starting from 1,4-dicyanobenzene via ionothermal method.

CTFs are only called and accepted as such if the temperature does not exceed 400 °C during the ionothermal synthesis. Even up to 400 °C the loss of nitrogen and the subsequent nitrogen deficit is quite high compared to the ideal structure. Above 400 °C nitrogen loss and deficit increase drastically such that the materials become essentially nitrogen-doped carbon materials. These materials must be compared to active carbon and no longer to CTFs that were prepared at 400 °C. Generally, at 600 °C higher surface areas and total pore volumes for the resulting “CTF” products are found than after comparable syntheses at 400 °C [1-3].

In PXRD, only two broad diffraction reflexes at around 8° and 22° 2θ, corresponding to the (100) and (001) planes, can be observed (Figure S1). Kuhn et. al. showed, that

the broad reflex at 26.1° 2θ corresponds to the (001) interlayer distance of 3.4 Å of the aromatic sheets [4]. In our case, the maximum of this broad reflex is shifted towards 2θ = 21.9°, which was explained by Bhunia et. al [5] as an increase of the layer distance between the triazine sheets to 4.1 Å.

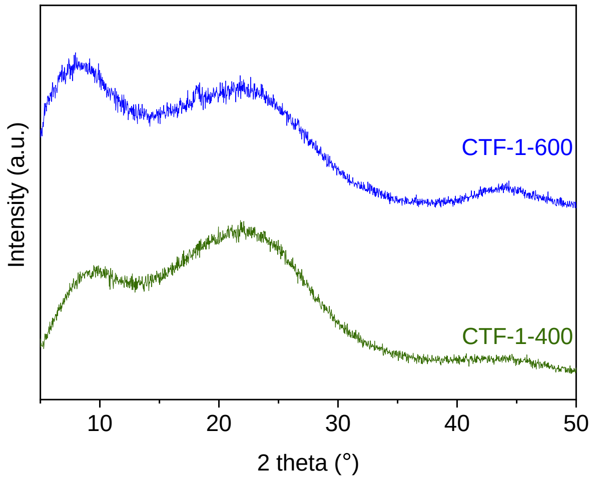

Figure S1: PXRD patterns of CTF-1-400 and CTF-1-600.

As a result of increased synthesis temperature, higher porosity and enhanced surface area have been observed for CTF-1-600 (Figure S2) as expected from earlier studies, where a surface area of 920 and 1750 or 2660 m^2/g (depending on the reaction time) was reported for CTF-1-400 and CTF-1-600, respectively [2]. The surface area for CTFs does not only vary with temperature but also with reaction time. The increase in surface area with temperature is a result of nitrogen loss and occurring defects due to the high temperature. This results in a higher pore volume and the formation of mesopores in the material [2].

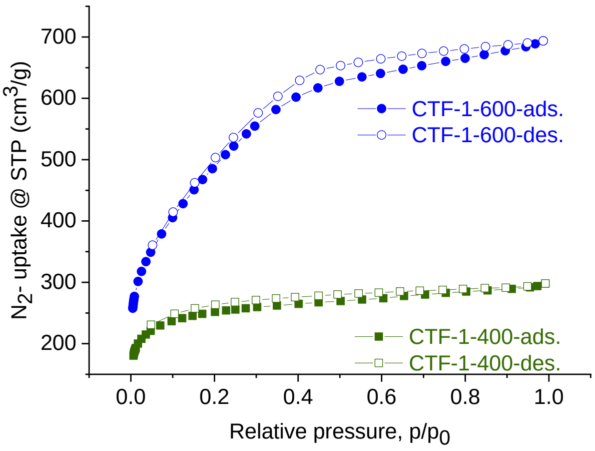

Figure S2: Nitrogen adsorption and desorption isotherms (at 77 K) of CTF-1-400 and CTF-1-600. See Table S2 and Figure S14 for the pore volumes and pore size distribution, respectively.

Elemental analyses yielded the expected C, H and N content (Table S1), in agreement with previous literature sources [2,5]. Increasing the synthesis temperature to 600 °C resulted in an increased C content and decreased N and H contents, which is explained by further linkage of the aromatic building blocks at higher temperatures, which occurs via [CN] and H_2 elimination [2].

Table S1: Elemental analysis of CTF-1-400 and CTF-1-600.[a]

	C [wt. %]	N [wt. %]	H [wt. %]	Rest [wt. %]	atom C/N
CTF-1 calculated*	74.99	21.86	3.15	—	4
CTF-1-400	75.92	14.54	2.75	6.79	6.1
CTF-1-600	79.27	9.43	1.09	10.21	9.8

[a]Calculation based on the idealized structure of CTF-1.

Thermogravimetric analyses under nitrogen atmosphere revealed the decomposition of the materials above 400 °C and 600 °C for CTF-1-400 and CTF-1-600, respectively. (Figure S3).

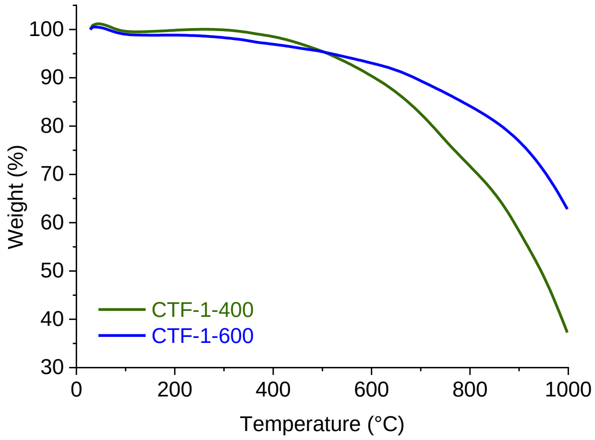

Figure S3: Thermogravimetric analysis (TGA) of CTF-1-400 and CTF-1-600 in the temperature range of 25–1000 °C under N_2 atmosphere with a heating rate of 10 K/min.

Scanning electron microscopy (SEM) showed a shard morphology with a wide particle size distribution for both CTFs (Figure S4) and slight impurities of chlorine and zinc have been found by energy dispersive X-ray spectrometry (EDX), which can be seen in Figure S5.

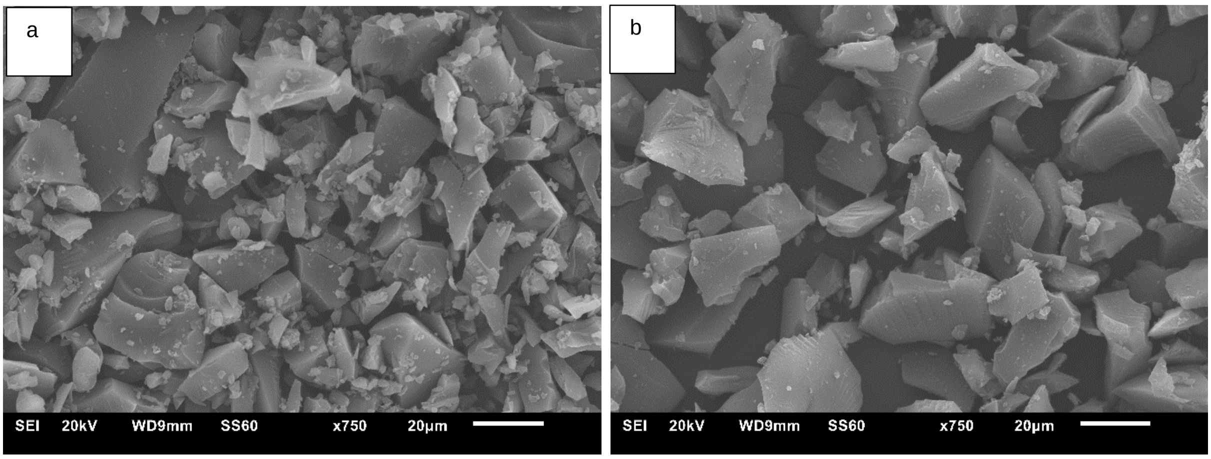

Figure S4: SEM images for a) CTF-1-400 and b) CTF-1-600.

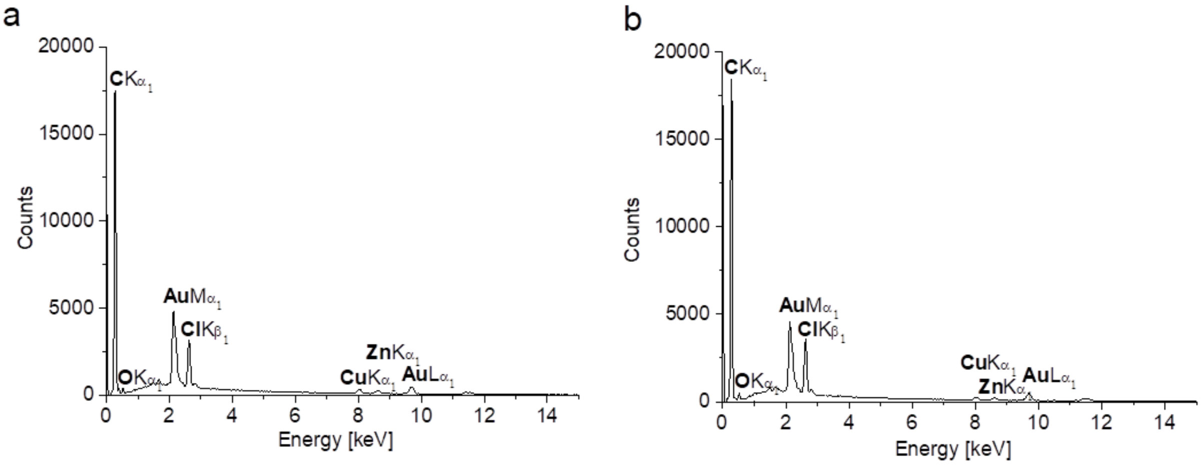

Figure S5: EDX analysis for (a) CTF-1-400 and (b) CTF-1-600.

Characterization of Ni/CTF-1 composites

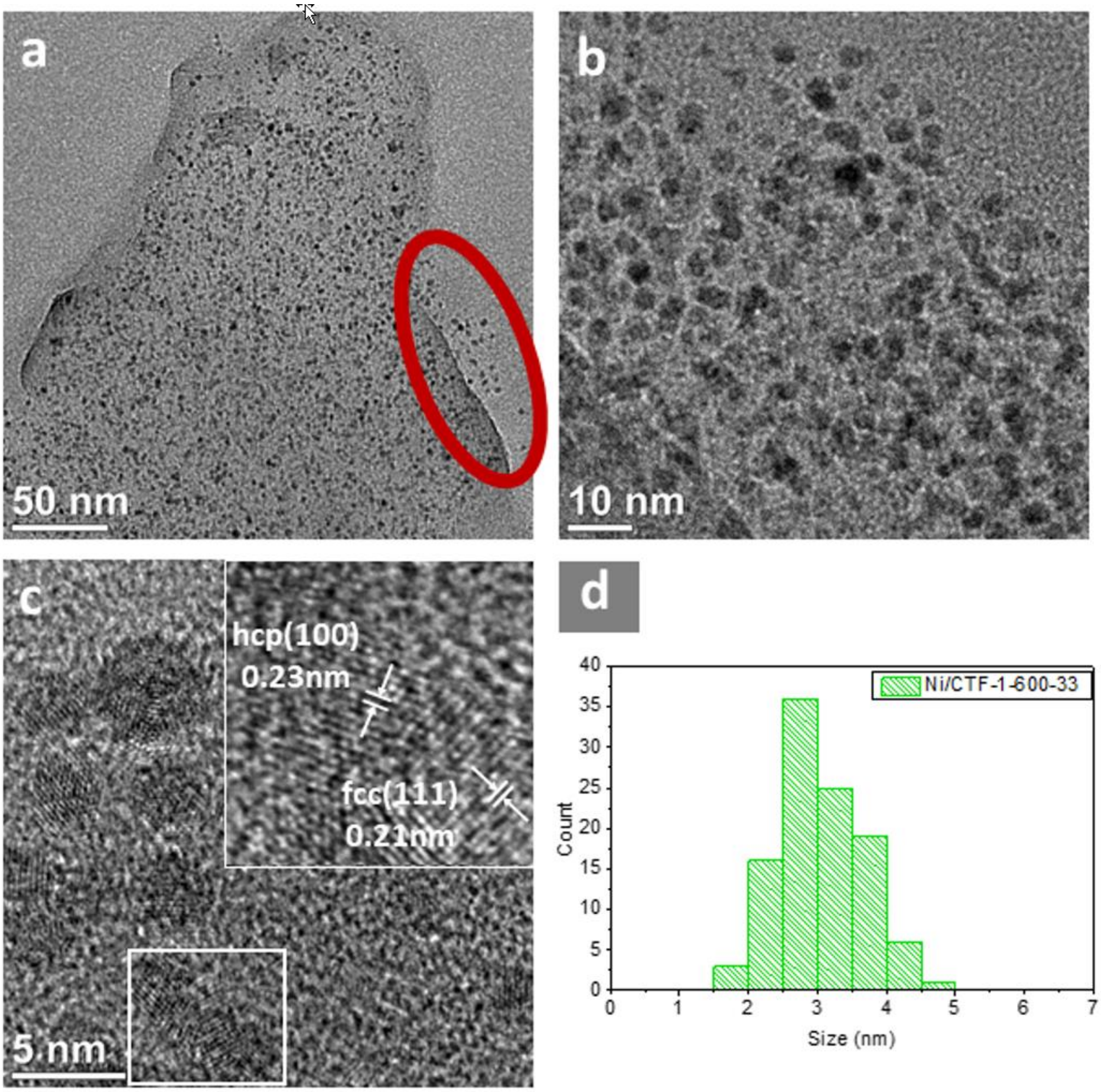

Figure S6: (a–c) TEM images of Ni/CTF-1-600-33 with (d) histogram giving the average diameter of 3.0 ± 0.6 nm. The red circle in (a) shows Ni NPs that lie in direct proximity to the support, having been “washed off” from the CTF to the carbon grid, probably at the time of sample preparation. This is an indication that at least some NPs are located on the surface.

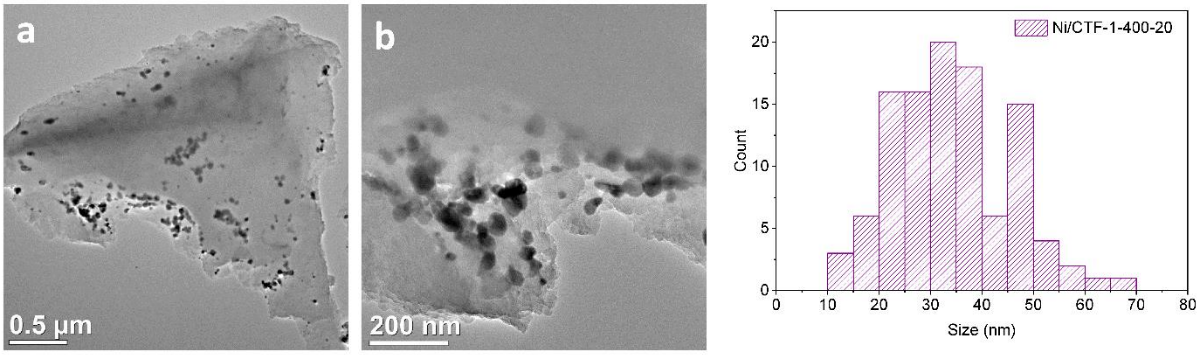

Figure S7: (a,b) TEM images of Ni/CTF-1-400-20 with histogram giving the average diameter of 35 ± 13 nm.

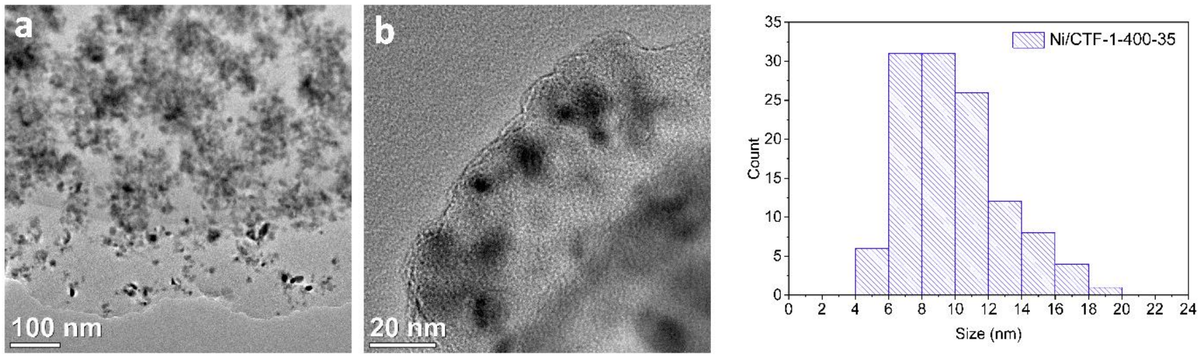

Figure S8: TEM images of Ni/CTF-1-400-35 with the average diameter of 10 ± 3 nm.

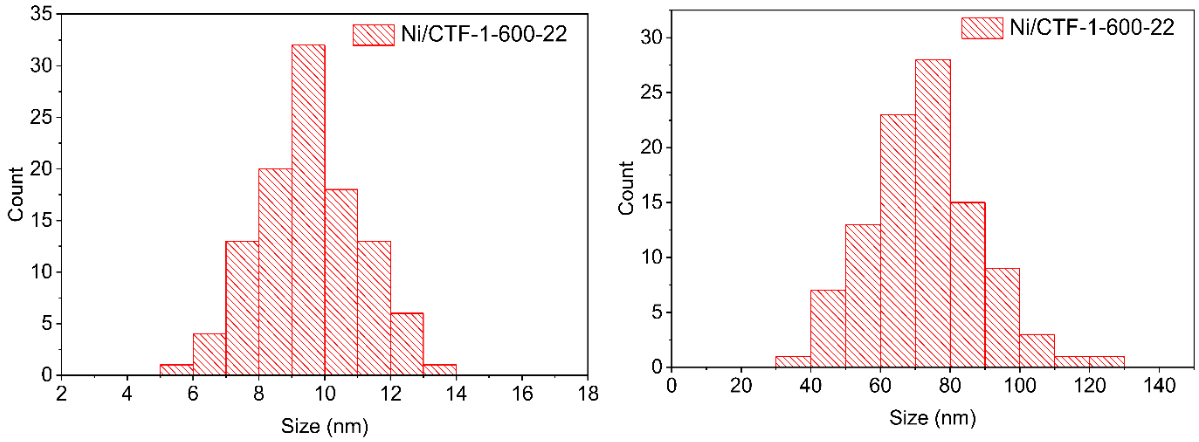

Figure S9: Nickel nanoparticle size distribution of Ni/CTF-1-600-22 with an average diameter of 10 ± 2 nm and aggregated nanoparticles with an average diameter of 72 ± 16 nm.

To further show the presence of particles on the surface, we present in Figure S10 TEM images with a “side view” of the materials so that the Ni NPs are imaged to stick out from the surface. Figure S10 clearly shows Ni nanoparticles that are sticking out over the edge of the CTF. This means at least some particles are at the surface. In Figure S10c, single nanoparticles are even somewhat bordered within the carrier but protrude with a part or a surface. This observation does not disprove that some particles might be encapsulated by the CTF. Additionally, XPS, which is a surface technique (Figure 5 and Figures S15–S18), confirms the presence of Ni on or very close to the surface of the CTF material.

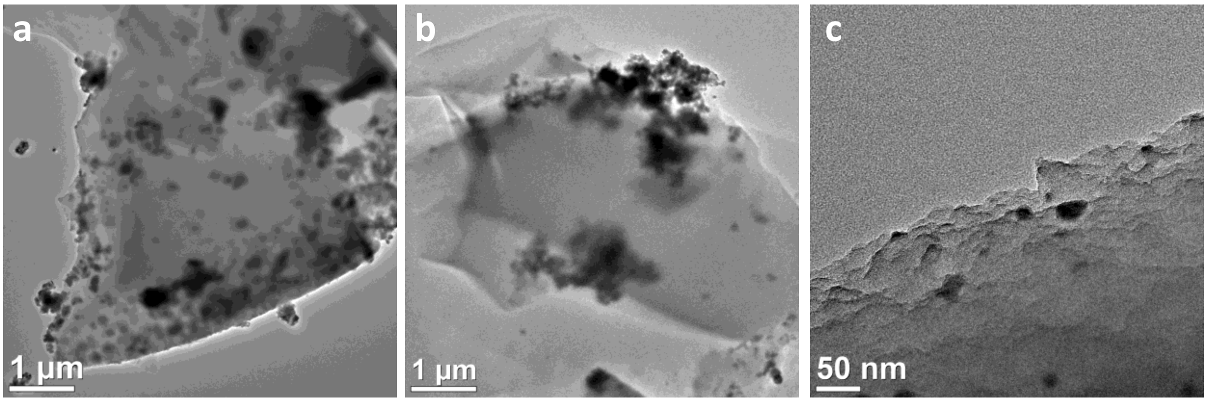

Figure S10: TEM pictures of (a) Ni/CTF-1-600-33 and (b,c) Ni/CTF-1-400-20.

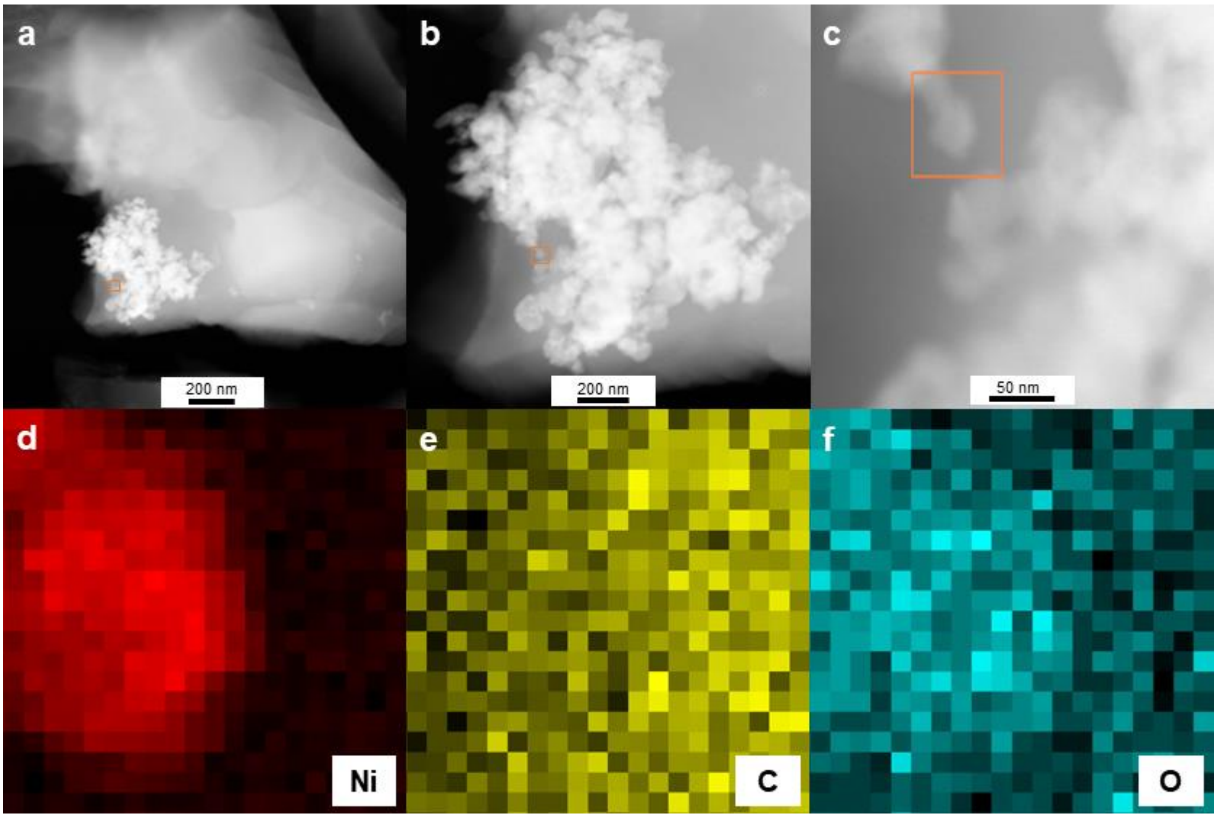

Figure S11: (a–c) STEM images at different magnifications and EDX elemental mapping of Ni/CTF-1-600-22, showing a CTF structure with supported Ni NPs. An accumulation of Ni NPs was selected and the elemental composition of the area inside the orange square was investigated. (d) Nickel is shown in red, (e) carbon is shown in yellow and (f) oxygen is shown in blue.

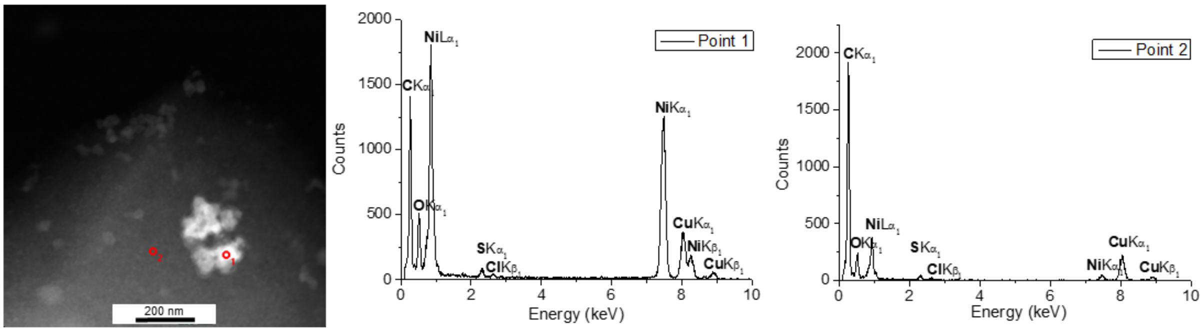

Figure S12: STEM image and point EDX analysis of a Ni NP agglomerate (point 1) and the CTF support (point 2) to verify that nickel is found not only as larger nanoparticles but also across the seemingly bare surface. An estimated atomic fraction of Ni of 64% is found, whereas 36% is found for O. This gives a ratio of Ni/O ≈ 1.8:1 (to be compared with Ni/O = 1:1 and $Ni/(OH)_2$ = 1:2, which indicates that a significant amount of Ni at point 1 is not oxidized (see the estimation details below).

Estimation of atomic Ni/O ratio

The Ni K lines were evaluated, not the Ni L line, since the latter has an unresolved overlap with the Cu L line and is therefore not reliable for concentration measurements. The Ni K and O K line intensities are measured after background subtraction. The background estimation was done by interpolation from neighboring channels, where no X-ray emission is expected. The composition of Ni and O was estimated using the Cliff–Lorimer method [6] for very thin foils, with k-factors of *k*(Ni K) = 1.508 and *k*(O K) = 1.889 [7]. The relative errors of the estimated atomic fractions and ratios are not better than 5% including systematic and statistical errors of measurement and evaluation. For the net values the difference of the two measurements was calculated and re-evaluated for mass fractions and from there to atomic fractions. By this, we assume, that the spectrum measured at point 1 also has contributions from the underlying CTF and that these contributions can be estimated from the counts measured at point 2.

Point 1 (particles above CTF):

- intensity (Ni K): 39000 +/- 1000

- intensity (O K): 8200 +/- 500

- atomic fraction Ni: 51%

- atomic fraction O: 49%

- ratio Ni/O : 1.0

Point 2 (CTF near the particles) :

- intensity (Ni K): 1200 +/- 200

- intensity (O K): 3600 +/- 300

- atomic fraction Ni: 7%

- atomic fraction O: 93%

- ratio Ni/O : 0.08

Net values for point 1, assuming the set of particles is on a CTF with identical Ni and O content:

- intensity (Ni K): 38000 +/- 1000

- intensity (O K): 4600 +/- 600

- atomic fraction Ni: 64%

- atomic fraction O: 36%

- ratio Ni/O : 1.8

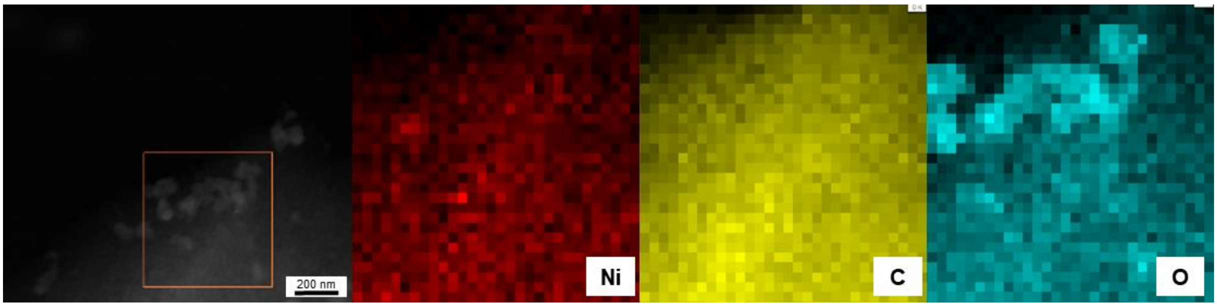

Figure S13: STEM image and EDX elemental mapping of Ni/CTF-1-600-22 showing nickel (in red), carbon (in yellow) and oxygen (in blue). The orange window in the STEM image displays the area of EDX mapping.

Porosity characterization

Table S2: Pore volumes of CTF and Ni/CTF materials.

Catalyst	CTF-1-400	Ni/CTF-1-400-20	Ni/CTF-1-400-35	CTF-1-600	Ni/CTF-1-600-22	Ni/CTF-1-600-33
Total pore volume V_{total} (cm^3/g)[a]	0.45	0.30	0.15	1.06	0.48	0.45
Micropore volume V_{mic} (cm^3/g)[b]	0.33	0.01	0.07	0.13	0.05	0.03
V_{mic}/V_{total}	0.73	0.03	0.46	0.12	0.10	0.06

[a]The total pore volumes were determined at p/p_0 = 0.95 from the adsorption branch for for pores ≤40 nm. [b]Micropore volume derived from t-plot analysis of the N_2 adsorption isotherm at p/p_0 = 0.2–0.5.

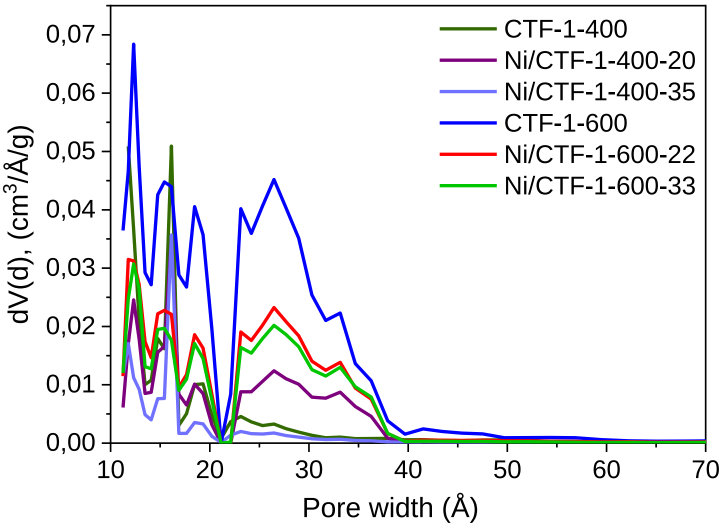

Figure S14: Pore size distribution from N_2 adsorption isotherm analysis (at 77 K).

X-ray photoelectron spectroscopy (XPS)

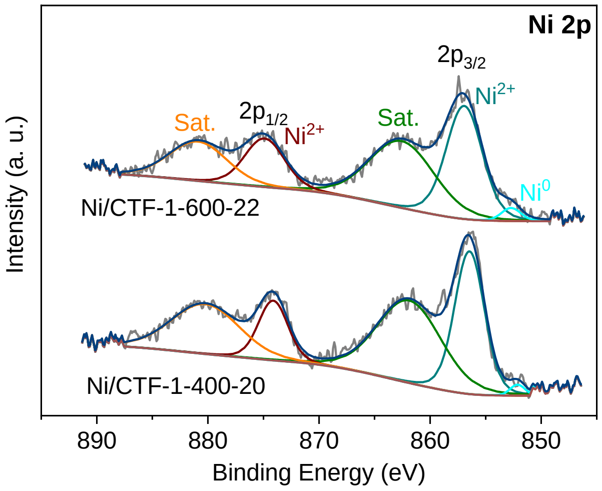

Figure S15: XPS measurement of Ni/CTF-1-400-20 and Ni/CTF-1-600-22 with the corresponding deconvoluted Ni 2p spectra.

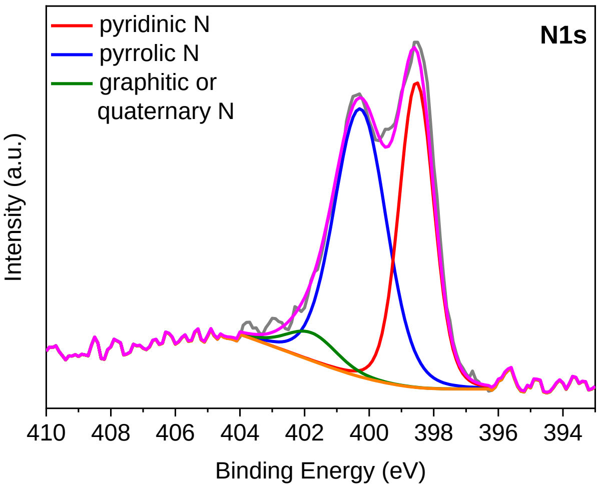

Figure S16: XPS measurement of CTF-1-400 with deconvoluted N 1s spectrum.

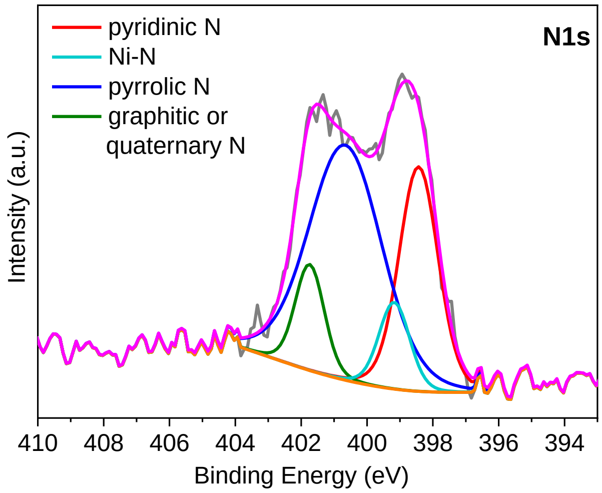

Figure S17: XPS measurement of Ni/CTF-1-400-20 with deconvoluted N 1s spectrum.

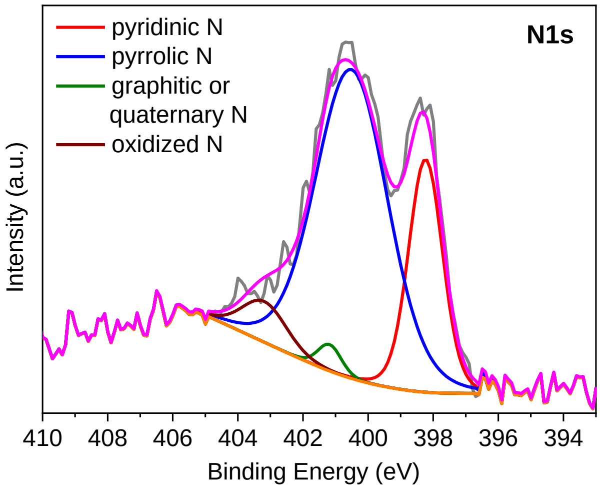

Figure S18: XPS measurement of CTF-1-600 with deconvoluted N 1s spectrum.

Tafel plots for OER and ORR

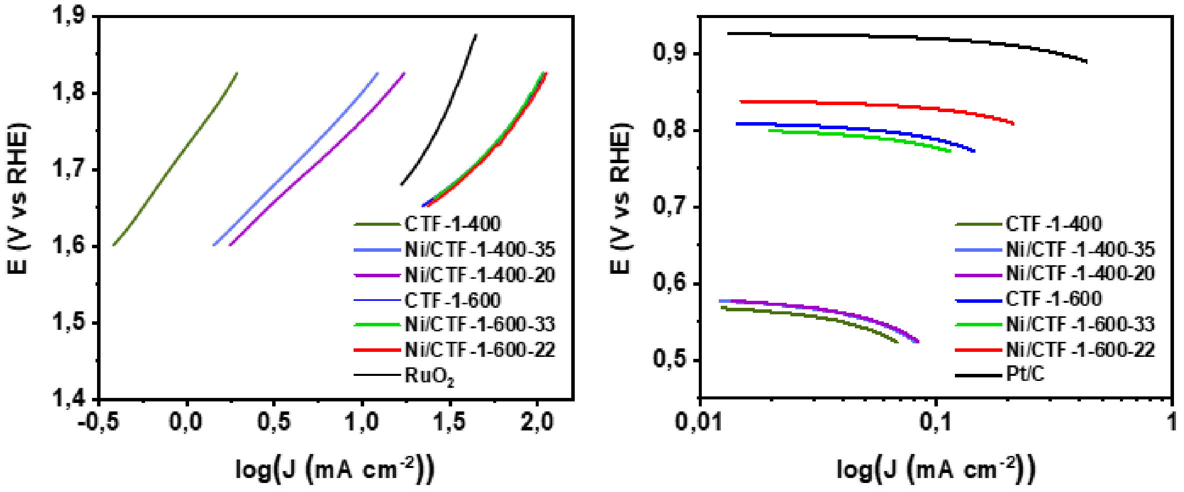

Figure S19: Tafel plots for OER (left) and ORR (right) results.

Table S3: Comparison of supported nickel/carbon material based OER catalysts.

Sample	Synthesis	Temperature (°C), time, atmosphere	Ni content (method)	KOH Electrolyte conc. (mol/L)	overpotential (at 10 mA/cm^2 vs. RHE) (mV)	Onset potential (V)	Ref.
Ni/CTF-1-600	Ionothermal, microwave rad.	230, 10 min, N_2	22 wt % (AAS)	1.0	374	1.51	This work
Ni@N-CNT-600[a]	MOF synthesis, Pyrolysis	600, 3h, N_2	7.3 atom % (XPS)	0.1	460	1.49	[8]
Ni@N-CNT-700	MOF synthesis, Pyrolysis	700, 3h, N_2	9.6 atom % (XPS)	0.1	—	1.59	[6]
Ni@N-CNT-800	MOF synthesis, Pyrolysis	800, 3h, N_2	21.2 atom % (XPS)	0.1	—	1.67	[6]
Ni/C[b]	Impregnation, pyrolysis	800, 1.5h, N_2	10.61 wt % (TGA)	0.1	420	1.59	[9]
Ni/NC[c]	Impregnation, pyrolysis	800, 1.5h, N_2	7.62 wt % (TGA)	0.1	390	1.56	[7]
Ni@graphene	MOF synthesis, annealing	600, 6h, Ar	74.10 wt % (EDX)	1.0	370	—	[10]

Ni@N-NC[d]	Pyrolysis of precursors	550, 3h then 700, 2h, Ar/N_2	not given	1.0	371	54	[11]
Ni@ketjenblack	Electrodeposition	not given	not given	1.0	340	—	[12]
Ni/carbon matrix	Complex grown on carbon, Pyrolysis	800, 5h, N_2	1 atom % (EDX)	0.1	390	1.52	[13]
fcc-Ni@N-C[e]	MOF synthesis, Annealing	500, 2h, Ar	69 wt % (ICP-MS)	1.0	360	—	[14]
hcp-Ni@N-C	MOF synthesis, Annealing	400, 2h, Ar	71 wt % (ICP- MS)	1.0	305	—	[12]
Ni/N-CNTs[a]	Annealing	500, Ar then 700, 2h, Ar	1.73 wt % (XPS)	0.1	590	—	[15]
MWCNTs	purchased	—	—	0.1	650	—	[13]
Ni@single layer graphene	Chemical vapor deposition	700, 20 min, 50% H_2/Ar	32.8 wt % (ICP)	1.0 (NaOH)	≈350	—	[16]
Ni@NC species[c]	Electrospinning, carbonization	800, 2h, N_2	14.5 wt % (TGA)	1.0	305	—	[17]

Ni-graphene film	Electrodeposition	50, 25 min	not given	0.1	392 @5mA cm^{-2}	1.56	[18]
Graphene	—	—	—	0.1	—	1.63	[18]
Ni@NC_x[f]	MOF Synthesis, Pyrolysis	600, 1h, N_2 then 800, 1h, N_2	25.2 wt % (ICP-OES)	0.1	—	1.56	[19]

[a]N-CNT = N-doped carbon nanotube, [b]C = carbon, [c]NC = nitrogen-doped carbon, [d]N-NC = nitrogen-doped nanocarbon

[e]N-C = N-doped carbon shells, [f]NC_x = graphitic layers coupled with a graphene sheet

References

1. Kuhn, P.; Thomas, A.; Antonietti, M. *Macromolecules* **2009,** *42,* 319–326. doi:10.1021/ma802322j
2. Kuhn, P.; Forget, A.; Su, D.; Thomas, A.; Antonietti, M. *J. Am. Chem. Soc.* **2008,** *130,* 13333–13337. doi:10.1021/ja803708s
3. Hug, S.; Stegbauer, L.; Oh, H.; Hirscher, M.; Lotsch, B. V. *Chem. Mater.* **2015,** *27,* 8001–8010. doi:10.1021/acs.chemmater.5b03330
4. Kuhn, P.; Antonietti, M.; Thomas, A. *Angew. Chem., Int. Ed.* **2008,** *47,* 3450–3453. doi:10.1002/anie.200705710
5. Bhunia, A.; Dey, S.; Bous, M.; Zhang, C.; Rybinski, W.; Janiak, C. *Chem. Commun.* **2015,** *51,* 484–486. doi:10.1039/C4CC06393G
6. Lorimer, G. W.; Al-Salman, S. A.; Cliff, G. The Quantitative Analysis of Thin Specimens: Effects of Absorption, Fluorescence and Beam Spreading in Developments. In *Electron Microscopy and Analysis*; Misell, D. L., Ed.; The Institute of Physics: Bristol and London, UK, 1977; pp 369–371.

7. k-factors for Ni K and O K are taken from the detector specific settings of the "TEM Image & Analysis" software, version 4.12, ThermoFisher Scientific, Eindhoven, Netherlands.
8. Han, H.; Chao, S.; Yang, X.; Wang, X.; Wang, K.; Bai, Z.; Yang, L. *Int. J. Hydrogen Energy* **2017,** *42,* 16149–16156. doi:10.1016/j.ijhydene.2017.05.043
9. Ren, J.; Antonietti, M.; Fellinger, T. P. *Adv. Energy Mater.* **2015,** *5,* 1401660. doi:10.1002/aenm.201401660
10. Ai, L.; Tian, T.; Jiang, J. *ACS Sustainable Chem. Eng.* **2017,** *5,* 4771–4777. doi:10.1021/acssuschemeng.7b00153
11. Zhang, X.; Xu, H.; Li, X.; Li, Y.; Yang, T.; Liang, Y. *ACS Catal.* **2016,** *6,* 580–588. doi:10.1021/acscatal.5b02291
12. Lee, S. Y.; Jung, H.; Chae, S. Y.; Oh, H-S.; Min, B. K.; Hwang, Y. J. *Electrochim. Acta* **2018,** *281,* 684–691. doi:10.1016/j.electacta.2018.05.170
13. Cui, C.; Ge, X.; An, T.; Li, B.; Wuu, D.; Tham, N. N.; Zhang, K.; He, Y.; Liu, Z. *Inorg. Chem. Front.* **2019,** *6,* 1873. doi:10.1039/C9QI00402E
14. Wang, C.; Wang, Y.; Yang, H.; Zhang, Y.; Zhao, H.; Wang, Q. *Small* **2018,** *14,* 1802895. doi:10.1002/smll.201802895
15. Liu, Y.; Jiang, H.; Zhu, Y.; Yang, X.; Li, C. *J. Mater. Chem. A* **2016,** *4,* 1694–1701. doi:10.1039/C5TA10551J
16. Cui, X.; Ren, P.; Deng, D.; Deng, J.; Bao, X. *Energy Environ. Sci.* **2016,** *9,* 123. doi:10.1039/C5EE03316K
17. Li, H.; He, Y.; Yang, Q.; Wang, J.; Yan, S.; Chen, C.; Chen, J. *J. Solid State Chem.* **2019,** *278,* 120843. doi:10.1016/j.jssc.2019.07.004
18. Pu, Z.; Liu, Q.; Asiri, A. M.; Sun, X. *J. Appl. Electrochem.* **2014,** *44,* 1165–1170. doi:10.1007/s10800-014-0743-6
19. Zhu, J.; Xiao, M.; Zhang, Y.; Jin, Z.; Peng, Z.; Liu, C.; Chen, S.; Ge, J.; Xing, W. *ACS Catal.* **2016,** *6,* 6335–6342. doi:10.1021/acscatal.6b01503
